# A Case Series and Literature Review of Alveolar Echinococcosis in Kashmir, India: An Emerging Endemic Zone for *Echinococcus multilocularis*

**DOI:** 10.3390/life14070794

**Published:** 2024-06-24

**Authors:** Mohammad Sultan Khuroo, Naira Sultan Khuroo, Ajaz Ahmad Rather

**Affiliations:** 1 Digestive Diseases Centre, Dr. Khuroo’s Medical Clinic, Kashmir, Srinagar 190010, India; naira_sultan@yahoo.com; 2 Department of Surgery and Registrar Academics, Sher-I-Kashmir Institute of Medical Sciences Medical College and Hospital, Bemina, Kashmir, Srinagar 190010, India; drajazrather@gmail.com

**Keywords:** echinococcosis, alveolar echinococcosis, hydatidosis, *Echinococcus multilocularis*, Kashmir, albendazole, praziquantel

## Abstract

A prospective study on 110 patients with echinococcosis at Dr. Khuroo’s Medical Clinic, Srinagar, Kashmir, India, from March 2019 to April 2024 identified 12 cases (4 males, 8 females; mean age of 46.58 ± 11.97 years) of Alveolar echinococcosis (AE). Two patients were detected through ultrasound examinations carried out for unrelated causes; one presented with features of liver abscess, and nine had pain in the right upper quadrant for a mean period of 2.2 ± 1.79 years. All had the liver as the primary organ involved, with 15 tumor masses of a mean maximum diameter of 9.22 ± 3.21 cm and volume of 426 ± 374.61 cm^3^. Tumors placed centrally had invaded vessels and the biliary tract in eight patients, and those placed peripherally had invaded the liver capsule and adjacent organs in nine patients. Histologic examination of liver biopsies or resected organs revealed necrotic lesions, calcifications, and granulomatous inflammation with slender, thin-walled vesicles of bizarre configuration that stained strongly eosinophilic with periodic acid Schiff. Two patients had segmental liver resections; one was treated with liver aspiration, while the other nine with advanced disease received chemotherapy with albendazole along with praziquantel. Patients showed clinical improvement on a median follow-up of 12 months (range 1 to 60 months); however, MRI T2-weighted images and ^18^F-FDG-PET-CECT scans in two patients showed active disease on follow-up at one and five years, respectively. A systematic review detected 146 cases of AE in India from 1980 to April 2024. Twenty cases were from foreign countries, mostly from Central Asian republics, and 118 (93.65%) of the remaining 126 Indian patients were permanent residents of Kashmir Valley. The disease affected a population of 79,197 residing in 22 villages from 5 border districts of the valley. These villages were either high in or adjacent to the Himalayan mountain range. Disease prevalence in the affected population was 146.47/10^5^ (males 131.53/10^5^ and females 163.18/10^5^) and the incidence was 12.41/10^5^/year (males 11.16/10^5^/year and females 13.81/10^5^/year). Possible causes of the emergence of AE are discussed, and future directions for research to face this challenge arebeen identified.

## 1. Introduction

Echinococcosis is a zoonosis caused by cestodes (tapeworms) of the genus Echinococcus [1]. Adult worms live in the alimentary canal of the definitive host and maintain their life cycle through the interaction between definitive (predator) and intermediate (prey) hosts. Based on genetic testing and specific host–parasite interactions, the genus Echinococcus includes nine species: five species under *E. granulosus sensu lato* (*E. granulosus sensu stricto*, *E. canadensis*, *E. ortleppi*, *E. felidis*, and *E. equinus*), *E. multilocularis*, *E. vogeli*, extremely rare *E. oligarthrus*, and the recently identified Tibetan species *E. shiquicus* [2,3]. Humans are accidental intermediate dead-end hosts. Human infections are known to occur with *E. granulosus sensu stricto*, *E. canadensis*, and *E. ortleppi*, causing cystic echinococcosis (CE), *E. multilocularis* causing alveolar echinococcosis (AE), and *E. vogeli*, and *E. oligarthrus* causing polycystic echinococcosis (PE) [4].

AE is caused by the larval stages of *E. multilocularis* and primarily affects the liver as an infiltrative malignant disease and is uniformly fatal if left untreated [5]. AE occurs in the northern hemisphere and is an evolving threat to public health. Around 18,235 new cases are recorded every year in the world, with a disease burden of 666,433 disability-adjusted years (DALYs) [6]. China accounts for 91% of such cases, and the disease is highly endemic in a few regions on the Tibetan plateau (Qinghai, Sichuan, and Tibet), wherein disease prevalence is >3.0% in the general population [7,8]. AE has been a public health problem in Northern Japan for over 40 years [9]. Recently, there have been significant upward trends in the occurrence of human infection in Europe [10].

India is in the northern hemisphere and borders several endemic regions of *E. multilocularis*. CE is endemic in several regions in India, such as Andhra Pradesh, Chennai, and Kashmir; however, AE is not endemic in India [11], and only a few isolated cases have been reported in the past [12,13,14,15]. There has been a rising trend of AE in Kashmir, India, recently [16,17]. Here, we report on a prospective study with 12 cases of AE seen at Dr. Khuroo’s Medical Clinic (DKMC) and perform a systematic review of cases of AE in Kashmir, India.

## 2. Material and Methods

### 2.1. Material

Dr. Khuroo’s Medical Clinic, located in Srinagar, the capital of Kashmir, is a tertiary center for gastrointestinal and liver diseases. It caters to patients from the Kashmir Valley, Jammu, Ladakh, and other parts of India. The clinic prospectively follows all patients with echinococcosis as per standard protocol [1,5]. Here, we report 12 cases of AE seen at the clinic over 5 years, from March 2019 to April 2024.

### 2.2. Methods

Serodiagnosis. Echinococcus hydatid serology IgG was performed by ELISA using *E. granulosus* hydatid fluid. This is the first-line test for immunodiagnosis of both CE and AE, with a diagnostic accuracy of 95% for AE [18,19]. *E. multilocularis*-specific ELISA using Em2 antigens was not available.

Imaging techniques. A spectrum of imaging tools was used with the following objectives: i. characterize the liver masses, ii. define the extension of tumor masses toward the liver hilum involving the vascular system and biliary tract, iii. extension toward the liver capsule, infiltrating the surrounding organs, iv. parasite viability and activity at the onset and on follow-up, and v. distant metastases [20,21].

Ultrasonography was used as initial imaging to screen patients for liver masses [8]. Ultrasound with Doppler was useful for picking up calcification, screening for biliary dilatation, and evaluating vascular involvement by tumor masses. Because of its availability, low cost, and safety, ultrasound was performed at every 3-month clinic visit to evaluate any interim changes in liver lesions.

Triple-phase contrast-enhanced computed tomography (CECT) with splenoporto-venography was performed in all cases [22]. For this, helical 5-mm axial slices throughout the abdomen were acquired pre- and post-intravenous contrast administration. Plain, arterial, venous (70 s), and delayed phase images were obtained. Sagittal reformatted reconstructions were also created. CT was used as a modality of choice for global evaluation of liver masses, including number, size, location, calcification, contrast enhancement, hilar extension to vascular and biliary structures, capsule and surrounding organs, and chest involvement, if any.

Contrast-enhanced magnetic resonance imaging (CE-MRI) with magnetic resonance cholangiopancreatography (MRCP) was performed using T1/T2 FSE, HASTE, in-phase out-phase, coronal, and Axial T2 with MIP reconstruction [23]. MRI was used to determine parasitic activity. T2WI was examined to visualize metacestode vesicles (microcysts) and liquefaction necrosis (large cysts). Lesions were classified into five groups, as proposed by Kodama et al. [24], and considered active (groups 1, 2, and 3) and inactive (groups 4 and 5) [25]. In addition, diffusion-weighted imaging (DWI) was obtained to calculate the apparent diffusion coefficient (ADC) of the masses [17]. MRCP images were obtained to evaluate the invasion or compression of the biliary tree by the lesion.

^18^F-FDG PET-CECT scans from vertex to mid-thigh were obtained. One hour after administration of 10 mCi (370 MBq) of ^18^F-fluorodeoxyglucose, delayed imaging was acquired on an integrated 16-slice PET/CT Siemens Biograph Horizon scanner [26]. CT was used for attenuation correction and anatomical localization. SUVmax was normalized to lean body mass using SUVmax LBM. This modality was chosen to assess and monitor disease activity by peripheral FDG uptake with high SUVmax values [27].

**Figure 1:**
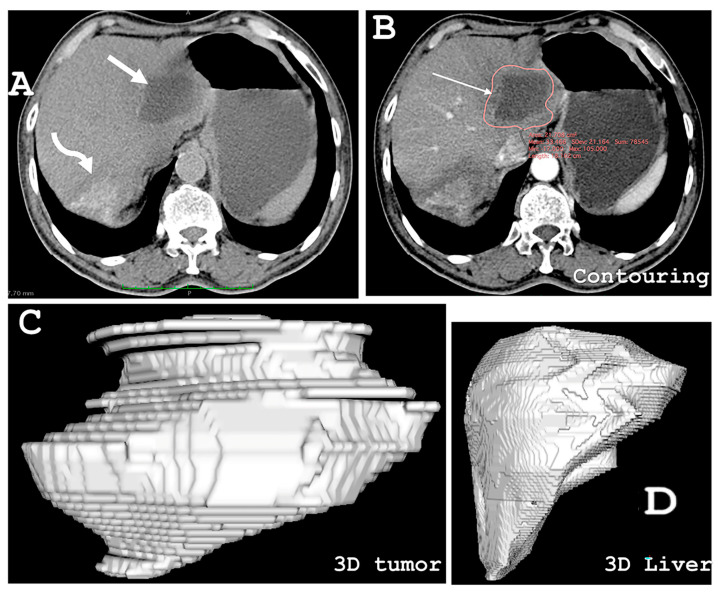
Liver volumetry. (**A**) Image of the liver extracted from the CD ROM with two masses (arrows). (**B**) Contouring the mass (red circle) in the left lobe using the dimensions of the slice (arrow). (**C**) Three-dimensional image of the tumor mass. (**D**) Three-dimensional image of the liver after liver contouring. Volumes: Left lobe tumor 174.2 cm^3^, right lobe tumor 80.3 cm^3^. Liver volume 1900 cm^3^. For details of the liver volumetry calculation, refer to Supplementary Document S1.

Tumor volumes (Figure 1). Tumor mass dimensions [length, breadth, and height] were measured using any of the liver imaging modalities. Tumor mass volumetry was performed using HOROS 3.3.5 software by contouring a series of axial images with slice thickness of 3–5 mm [28]. After selecting all the regions of interest (ROIs), the software automatically calculated the volume by multiplying surface and slice thickness and adding individual slice volumes. Before recommending resections for large liver masses, the future liver remnant volume (FLRV%) was evaluated. (For details, refer to Supplementary Document S1) [29].

Histology. The histological examination of tissues obtained through a needle, laparoscopic-aided biopsy, or resected specimens was performed. The tissue was fixed in 4% formalin and paraffin-embedded. Tissue sections (2 to 3 µm) were stained with hematoxylin and eosin (H&E), reticulin, and periodic acid-Schiff (PAS) [30].

Diagnosis. The diagnosis was initiated in every case following abnormal imaging findings, either carried out for unrelated causes or precipitated by symptoms. All cases had a critical review of findings at serology and imaging. The diagnosis was established based on histological findings of liver tissue obtained from needle liver biopsies or resected liver specimens. Based on the characteristics of the liver masses, their extension toward the hilum and involvement of the vascular and biliary tracts, extension to the liver capsule and surrounding organs, and distant metastases, each patient had PNM classification and staging as per the WHO informal working group on echinococcosis [31]. Management was determined by the PNM classification and stage of the disease, the metabolic activity of the parasite, and the possibility of complete resection of liver lesions [4,32]. All patients with active disease received albendazole [33,34] with or without praziquantel therapy [35,36].

Literature review. A systematic review was performed to document all cases of AE in Kashmir, India. The protocol was established under updated PRISMA guidelines 2020, which consisted of a 27-item checklist and a 4-phase flow diagram [37] (Supplementary Document S2). We have reported on several systematic reviews and meta-analyses under PRISMA guidelines [38,39]. A patient was accepted to have AE based on clinical, serological, imaging, and histological proof of *Echinococcus multilocularis*. For the purposes of the systematic review, three search methods were used, namely:

A. Published cases of AE: A literature search was conducted for published cases of AE from India. The PubMed database was searched using keywords such as alveolar echinococcosis, *Echinococcus multilocularis*, alveolar hydatid, and India. The search was expanded through several areas, including other search engines (EMBASE and Google Scholar), personal contacts, and a hand search of conference abstracts and dissertations. Duplicate articles were found in the library menu on EndNote 21 and removed. Abstracts were screened to select articles that dealt with case reports, case series, and case studies of AE from India. Two authors (MSK and NSK) independently conducted a full-text review of selected articles and studied cases published to confirm the inclusion criteria of AE. Any discrepancies were sorted out by consensus.
B. Cases of AE from DKMC: The systematic review included all cases of AE registered in the clinic from March 2019 to April 2024.
C. Cases retrieved from other medical centers: We approached various clinical units, liver transplant centers, imaging units, pathology departments in Kashmir, and several tertiary care hospitals in North India. A request was made to give us data on cases of AE registered and managed under their care but not published. We received a positive response from several units, including a list of patients with AE. This list was analyzed for duplications. Two authors (MSK and NSK) independently reviewed the list to confirm the inclusion criteria.

A demographic data table was prepared for all cases of AE recovered from the three search methods. We searched for the nationality of all cases. For Indian patients, we recorded residences from Kashmir and other parts of India. The residential locations and affected populations of Kashmiri AE cases seen at DKMC and retrieved from other medical centers were carefully identified. The prevalence and incidence rates were determined based on the total numbers and the affected populations [40]. The burden of disease of AE in Kashmir, India, was calculated using disability-adjusted life years (DALYs), which is the sum of years of life lost owing to premature mortality (YLL) and years of healthy life lost owing to disability (YLD) [41]. To calculate the YLL, we assumed the disease was fatal within 8 years, and for YLD, a disability weight of 0.200 was applied, as suggested in the earlier study [6]. One DALY represented the loss of the equivalent of one year of full health.

## 3. Results

**Figure 2:**
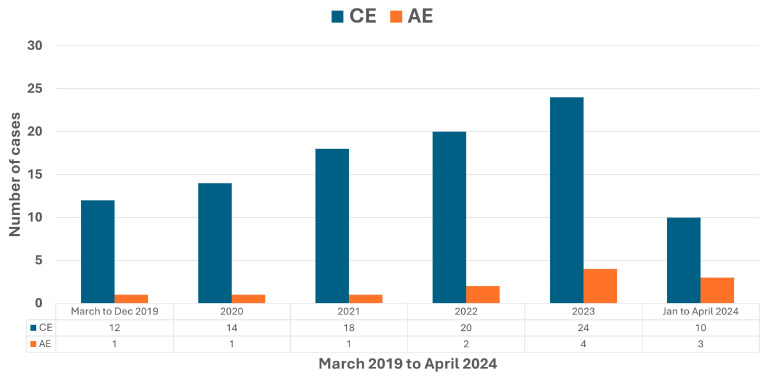
Year-wise distribution of cases of cystic echinococcosis (CE) and alveolar echinococcosis (AE) registered and prospectively followed at Dr. Khuroo’s Medical Clinic from March 2019 to April 2024.

From March 2019 to April 2024, 110 patients of echinococcosis were registered and followed at our clinic, including 98 cases of CE and 12 cases of AE. The year-wise distribution of CE and AE cases is shown in Figure 2. The age of patients with AE at presentation was 46.58 ± 11.97 years (95% CI 38.98 to 54.19). All patients were residents of Kashmir Valley, and none came from Jammu, Ladakh, or other parts of India. The patients belonged to tribal populations and lived either within the low mountainous regions of Kashmir (*n* = 4) or border areas of the valley adjacent to mountainous ranges (*n* = 8). The livelihood of these tribal populations was closely linked to forests, and they would often visit the forests over the years. None of the patients were long-term dog owners. No patient had traveled to endemic areas of AE. Apart from a hypothyroid state in one patient, none of the patients had known co-morbid disease, immunocompromised status, or solid organ transplant.

Presentation (Table 1). Two patients were detected during routine ultrasound examinations for unrelated causes, while the remaining ten were symptomatic. The patients complained of pain in the right upper quadrant (*n* = 10), icterus (*n* = 2), generalized aches and pains with arthralgia (*n* = 1), and severe fatigue (*n* = 1). The duration of illness was less than three months in two patients, three months to one year in three patients, and more than one year in the remaining five patients, with a mean of 2.20 ± 1.79 years [95% CI 1.09 to 3.30]. Physical examination in seven patients revealed firm to hard hepatomegaly with rounded margins and irregular surfaces. One patient had tender hepatomegaly, intercostal tenderness, fever, jaundice, and leukocytosis with elevated serum bilirubin and liver enzymes suggestive of liver abscess. Physical examination was unremarkable in the remaining four patients. Complete blood counts and coagulation profiles were within normal limits. Liver function tests were normal in six patients and showed elevated serum alkaline phosphatase in five. Hepatitis B surface antigen (HBsAg), hepatitis C antibodies (anti-HCV), and HIV-1 and II antibodies were non-reactive. Tumor markers, namely AFP, CEA, and CA19-9, were within normal ranges. Hydatid serology was strongly reactive in all cases.

Liver lesions. Based on the findings of multiple body imaging techniques, the liver was the primary disease site in all patients. It showed up as single or multiple voluminous infiltrative hepatic masses with no clear margins between lesions and healthy parenchyma. In all, 15 liver masses were detected. Nine patients had a single liver mass, while three patients had two masses each. A single mass was placed in the right lobe (in various segments) in seven patients and the left lobe in one patient. Two patients had one mass each in the right and left lobes. One patient had a multifocal lesion in the right lobe, and one patient had a large mass extending into both the left (segment IVB) and right (segment V) lobes. Liver segmental (Couinaud classification) involvement was as follows: two segments were affected by a single lesion in four patients, three segments in four patients (single lesion in three and multifocal lesion in one), and two lesions in each patient in segments 4 and 5, respectively. One lesion was <5 cm in maximal diameter, eight were 5 to ≤10 cm, five were 10 to ≤15 cm, and one was ≥15 cm, with a mean maximal diameter of 9.22 ± 3.21 cm (95% CI 7.59 to 10.84 cm). As determined by volumetry, the tumor volume was 426 ± 374.61 cm^3^ (95% CI 236.47 to 615.62 m^3^) (Figure 1).

**Figure 3:**
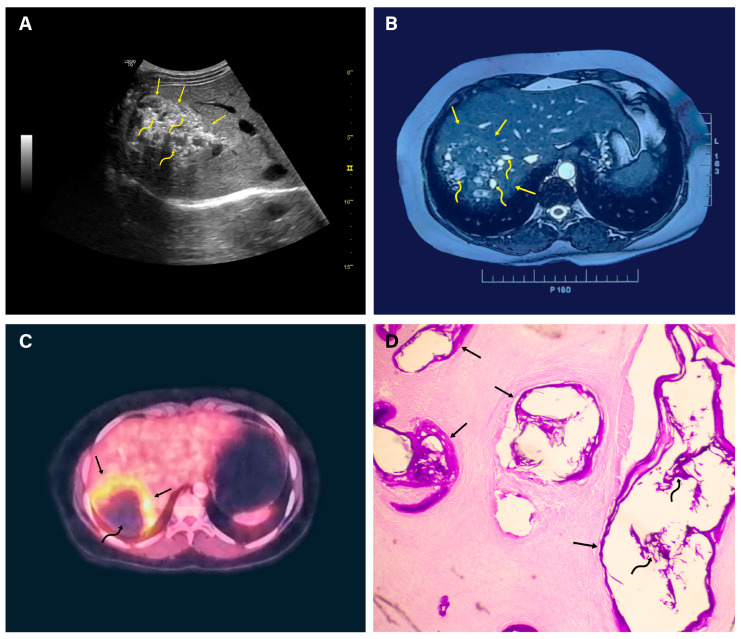
Alveolar echinococcosis. (**A**) Ultrasonography revealed a 7.0 × 6.7 cm lesion (straight arrows) in hepatic segment VII, with variable echo pattern and diffuse internal microcalcification (curved arrows). (**B**) T2W1 magnetic resonance imaging showed a liver lesion (straight arrows) containing numerous hyperintense microcysts (curved arrows) (Kodama type 1). The portal venous system, the hepatic veins, and the biliary ducts were not involved. (**C**) Positive emission tomography revealed fluorodeoxyglucose uptake at the periphery of the lesion (straight arrows) and no uptake in the center (curved arrow). (**D**) A laparoscopic guided liver biopsy of the mass revealed a fibrotic lesion containing numerous cysts, with laminar membranes stained intensely with periodic acid-Schiff stain (straight arrow). Diffuse dystrophic calcification within the cysts (curved arrows) and stroma. Hydatid hooklets were not seen in the lesion.

The appearances on imaging techniques were determined by the physical characteristics of the lesions, including their solid component, the extent of necrosis, the degree and pattern of calcification, the presence and extent of microvesicles, and the invasive potential of the tumor. On ultrasound, ten lesions appeared as heterogeneous echogenic masses with irregular borders, while the remaining five lesions had central anechoic components with thick echogenic walls. Doppler ultrasound did not reveal any areas of increased vascularity within or around the tumor. CE-CT yielded better characterization of lesions, which appeared as irregular marginated hypodense lesions or lesions with heterogeneous density with low attenuation values, varying from 30 to 50 HU. Post-contrast CT did not reveal any enhancement of the lesions. However, contrast enhancement was occasionally seen in the lesion’s periphery in a few cases. Of the 15 lesions, 11 showed calcification, as seen on ultrasound, and were better defined on non-contrast CT scans. It was speckled throughout in six lesions and amorphous in appearance in five. MRI depicted large lobulated masses with hypointense signal intensity on a T-1 weighted sequence. T-2 weighted sequences revealed hyperintense microvesicular cysts with no hypointense solid component (Kodama type 1) in one lesion, heterogenous signal intensity mass with hypointense solid and hyperintense cystic components (Kodama type 2) in five lesions, a large central hyperintense cystic lesion with a peripheral thick-walled solid hypointense component (Kodama type 3) in four lesions, a large hypointense solid mass with no hyperintense cystic component (Kodama type 4) in three lesions, and a large hyperintense cystic mass with no solid component (Kodama type 5) in two lesions. Post-contrast T1-weighted images revealed no enhancement of the lesion. DWI revealed free diffusion characterized by a high signal on ADC ranging from 1.39 × 10^−3^ mm^2^/s to 2.3 × 10^−3^/s. Six patients with eight liver lesions had ^18^F-FDG-PET-CT studies to evaluate disease activity. The lesions had a central hypometabolic (necrotic) component and rim of intense FDG avidity (SUVmax of 3.41 to 6.43) in the periphery (Doughnut sign) (Figure 3).

**Figure 4:**
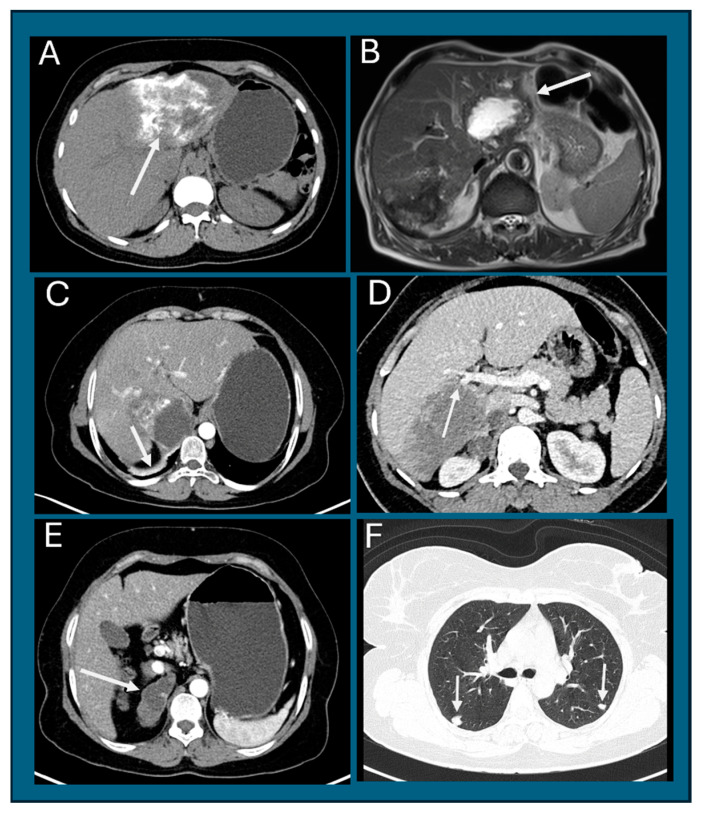
Alveolar echinococcosis. (**A**) NCCT. A large hypodense lesion in the left lobe invades the liver capsule with dense tumor calcification (arrow). (**B**) MRI. T2WI. Two tumor masses. The left lobe mass is hyperintense with a hypointense periphery, with involvement of the gastrohepatic ligament (arrow). The right lobe mass is hypointense, with involvement of the liver capsule. (**C**) CECT. Tumor mass in segment IV, with involvement and thickening of the right crura of the diaphragm (arrow). (**D**) CECT. Hypodense mass in the right lobe of the liver blocking a branch of the right portal vein (arrow). (**E**) CECT. Tumor mass in the right adrenal gland (arrow), with lymph nodes around IVC. (**F**) CT chest. Two nodular opacities in the right and left lungs (arrows).

**Figure 5:**
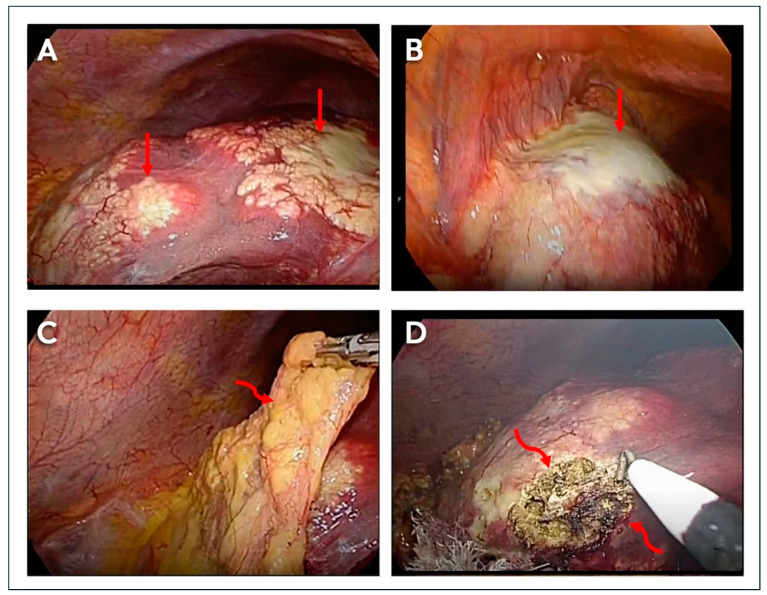
Alveolar echinococcosis with infiltration into the liver capsule, diaphragm, and omentum. The patient had a liver mass in the right lobe and another mass in the left lobe. Laparoscopy: (**A**) Right lobe of the liver with extensive yellow, cake-like, cheesy material (straight arrows) covering the liver capsule. (**B**) Left lobe of the liver with yellow cheesy material (straight arrow), liver capsule, and diaphragm. (**C**) Omentum with infiltration by yellow cheesy material (curly arrow). (**D**) Cut surface of the right lobe showing the tumor mass (curly arrows) with tiny, sponge-like cysts (microvesicles).

Tumor invasion and metastases (PNM classification). The tumors had a strong invasive potential (Figure 4). Tumors in three patients were placed peripherally within liver lobes, encasing segmental branches of the portal vein, bile duct, and hepatic artery (PNM P1). Tumors were placed centrally with proximal vascular and biliary involvement of one lobe in three patients (P2), both lobes in one patient (P3), and extending along the vessels and biliary tree in four patients (P4). Tumors had invaded the liver capsule and involved adjacent organs in nine patients (PNM N1). The adjacent organs included the right hemidiaphragm (*n* = 4), right adrenal gland (*n* = 4), omentum (*n* = 1), gastrohepatic ligament (*n* = 1), gallbladder (*n* = 1), stomach and duodenum (*n* = 2), and hilar lymph nodes (*n* = 2) (Figure 5). Lung metastases were seen in two patients and presented as multiple nodules of 3 mm to 1.5 cm (PNM M1). One of the patients had lymphadenopathy in the right hilum of the lung. Based on PNM classification, three patients had stage 1 disease, four had stage IIIb disease, and five had stage IV disease.

**Figure 6:**
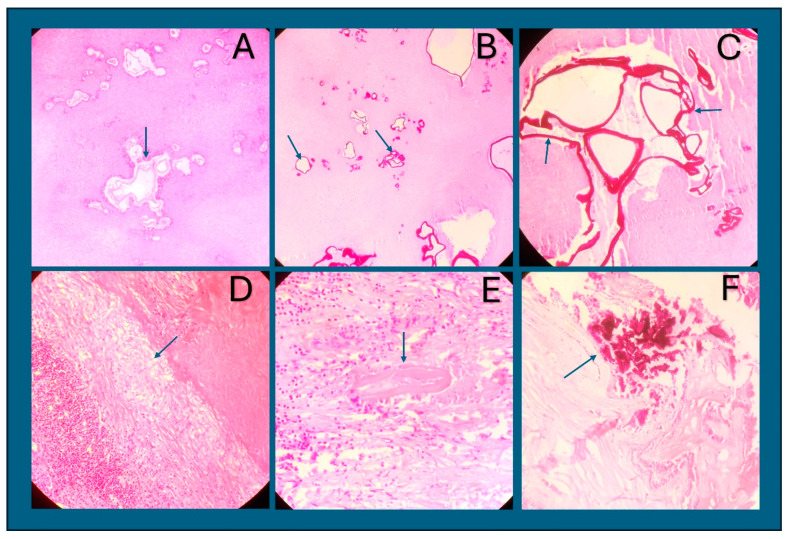
Alveolar echinococcosis. (**A**) HE × 100. Extensive necrosis with pale-pink laminated membranes of vesicles (arrow). (**B**) PAS × 100. Numerous vesicles of bizarre configuration and tubular growth patterns, with slender laminated membranes, strongly stained with PAS (arrows). (**C**) PAS × 200. A close-up view of laminated membranes of vesicles showing deep-violet PAS staining (arrow). (**D**) PAS × 100. Extensive necrosis with granulomatous reaction (arrow). (**E**) PAS × 100. A collapsed laminated membrane of a vesicle (arrow) with surrounding dense inflammation. (**F**) PAS × 100. Dense tissue calcification stained red (arrow).

Histology. The histology revealed poorly demarcated necrotic lesions containing numerous characteristic slender, thin-walled small vesicles of bizarre configuration. (Figure 6) The membrane of the vesicles stained weakly with H&E and strongly eosinophilic with PAS. The vesicles contained fluid-like material, which lacked protoscoleces. The lesions had no fibrous capsule, and a granulomatous cell reaction zone existed in the periphery, consisting of epithelioid cells, macrophages, fibroblasts, lymphocytes, plasma cells, and eosinophils. The lesions showed calcification, ranging from scattered nodular to diffuse and plaque-like patterns.

Treatment and Outcome. Of the three patients with early disease (stage I), two had segmental curative liver resections for excision of the tumor followed by albendazole therapy at 10 mg/kg/day and were free of disease at follow-up over one year. The third patient declined surgery, was on chemotherapy, and was lost to follow-up after one year. One patient with an infected necrotic cyst had percutaneous aspiration of the abscess along with broad-spectrum antibiotics followed by surgical excision of the necrotic material and long-term albendazole therapy. The patient has been doing well for two years on drug therapy. Eight patients with advanced disease (stage IIIb and stage IV) were treated with albendazole at 10–15 mg/kg/day plus praziquantel at 600 mg per day. Three patients have completed more than one year of follow-up. All patients had improved RHQ pain, regression in liver size on clinical examination, and repeat ultrasound carried out at three months showed progressive regression in the dimensions of the liver masses. None of the patients show disease progression. Repeat MRI examinations at one year in one patient and MRI with 18F-FDG-PET-CT at five years in another patient continued to show radiological evidence of active disease.

## 4. Systematic Review

**Figure 7:**
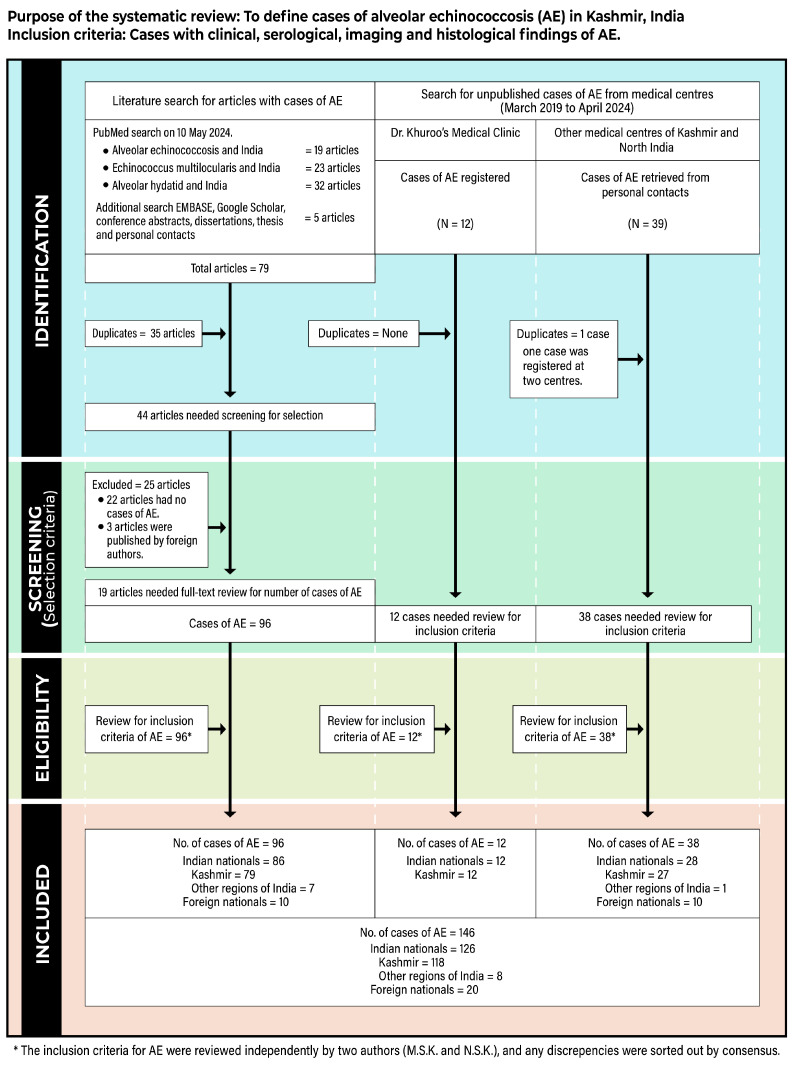
Flow diagram of the systematic review, as per PRISMA guidelines.

For the purposes of the systematic review, the three search methods revealed the following results (Figure 7; Table 2):

- Published cases of AE: The primary literature (PubMed) search revealed 74 articles. The expanded search (EMBASE, Google Scholar, and others) identified five more articles. Of the 79 articles, 35 duplicate articles were found in the library menu on EndNote 21 and removed. The abstracts of the remaining 44 articles were screened to identify articles that dealt with case reports, case series, and case studies of AE from India. Of these, 25 articles were excluded, as 22 included no cases of AE and 3 included cases of AE by authors from other countries. A full-text review of the remaining 19 articles identified 96 cases of AE. Ten cases were foreign nationals; 79 were from Kashmir Valley, and 7 were from other regions of India.
- Cases of AE from DKMC: DKMC prospectively follows echinococcosis cases from March 2005. Twelve cases of AE, not published previously, were registered in the clinic from March 2019 to April 2024. All cases were from Kashmir valley.
- Cases retrieved from other medical centers in Kashmir and North India: Several clinical units submitted a list of 39 cases. One case was registered in two centers, giving a final list of 38 cases. Of these, 10 were foreign nationals, 27 were from Kashmir Valley, and 1 was from another part of India.

Cases of AE included in the systematic review. Based on searches A, B, and C (Table 2), a list of 146 cases of AE in India was found. These included 20 foreign nationals (Central Asian Republics 18, Iraq 1, and Peru 1) and 126 Indian nationals, of which 118 (93.65%, 95% CI 93.07 to 94.23%) were from Kashmir Valley and 8 were from other parts of India. Of the eight patients, three were Indian Soldiers who possibly could have been infected during their postings in the Himalayan regions of Kashmir. Of the 146 cases, one was a 7-year-old child from Iraq, while all others were adults aged 40 to 60. There were 64 males and 82 females. The primary organ involved was the liver in 143 patients, while one patient each had primary disease in the lung, brain, and spleen/peritoneum. The majority of patients had stage IV disease. Living donor liver transplant was performed in 10 cases, all nationals from central Asia. Surgery, including segmental and lobar resections and reconstructions of hepatic vessels and bile ducts, was carried out in 34 cases. Four deaths were reported. Three patients died of disease recurrence after an incomplete surgical procedure, and one child died of advanced lung disease caused by AE.

**Figure 8:**
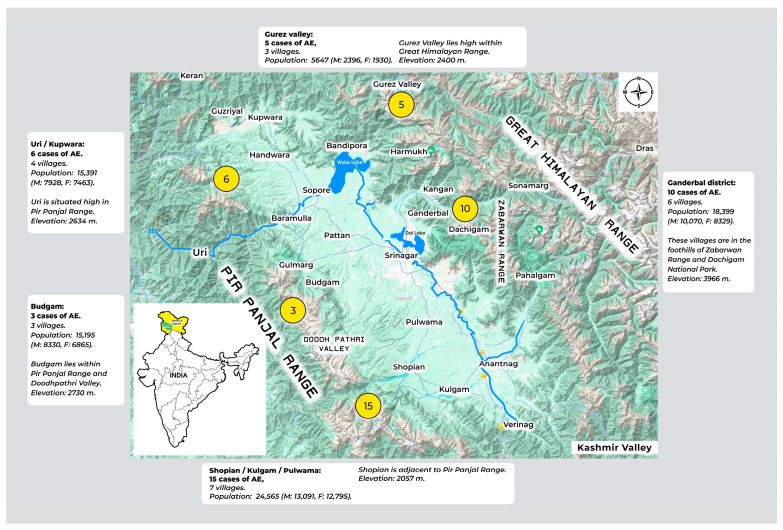
A map of Kashmir Valley with surrounding mountainous ranges shows the origin of 39 alveolar echinococcosis cases and their geographical characteristics.

Distribution of cases of AE from Kashmir Valley. This information was available in 39 Kashmir patients (12 cases from DKMC and 27 registered in various medical centers). The cases originated from 22 villages from 5 border districts with a population of 79,197 (M 41,815, F 37,382). These villages are located high in the mountains surrounding the valley (Great Himalayas, Pir Panjal ranges, and Zabarwan) or their foothills. There were no reports of AE from the valley bottom (Figure 8).

Incidence and Prevalence of AE in Kashmir, India. The total number of cases reported from Kashmir was 118 (M 56, F 62) over 12 years (March 2012 to April 2024), originating from a select population of 79,197 (M 41,815 and F 37,382), with two reported deaths. The incidence of cases per year was 9.83, with disease prevalence in the affected population of 146.47/10^5^ (males 131.53/10^5^ and females 163.18/10^5^) and an incidence of 12.41/10^5^/year (males 11.16/10^5^/year and females 13.81/10^5^/year). Based on standard assumptions [6], we estimated the total number of DALYs for AE in Kashmir, India, at 3100.56 (males 1457.8 and females 1684) with YLL of 2822.56 (males 1304.8 and females 1488.00) and YLD of 278 (males 153 and females 196).

## 5. Discussion

The present study identified Kashmir, India, as a high endemic zone for AE. AE in Kashmir occurred in a select tribal population with 9.83 cases per year, a disease prevalence rate of 146.47/10^5^, and an incidence rate of 12.41/10^5^/year. We estimated the total number of DALYs for AE in Kashmir, India, at 3100.56, with YLL of 2822.56 and YLD of 278. Table 3 shows the global occurrence of AE. AE is highly endemic in seven counties in Northwest China, which accounts for over 90% of the global disease load [6]. Russia is another endemic zone for AE, stretching from Eastern Europe to Siberia [9]. The Central Asian Republics and Turkey have a very high disease load [56,57,58]. AE has been a public health problem in Northern Japan for over 40 years [59]. The annual incidence of human infection in Europe is low (0.15 to 0.21 × 10^5^/year). However, a high disease load is reported in several European countries [60,61]. Most *E. multilocularis* infections from North America are reported in animal hosts, and only a few cases of human AE have been reported [62,63]. Globally, AE has a median of 666,434 DALYs per annum (CI 331,000–1.3 million), with China contributing 95% of the YLL [6]. Based on these data, the endemicity of AE in Kashmir resembles that of the Central Asian Republics.

How reliable are the data related to disease load in Kashmir? The clinical profiles of our patients with AE were distinctive. The liver was the primary site of involvement in all 12 cases and presented as liver masses on abdominal ultrasonography [1,66]. Ultrasound is cheap, noninvasive, widely available, and accurate in detecting liver masses. Thus, it is unlikely that patients with AE in an endemic area like Kashmir will stay undetected. In fact, two of our patients had liver masses detected during abdominal ultrasound examinations carried out for unrelated symptoms. However, benign-looking liver masses detected during ultrasound examinations may be misinterpreted as liver cysts or hemangiomas, and those with an invasive nature as hepatocellular cancer. Kashmir is an endemic zone for parasitic diseases of the liver and biliary tract [67,68], and clinicians always consider echinococcosis as a possibility in any liver mass [1]. On this basis, we believe the incidence rates of AE in Kashmir are fairly accurate.

AE is emerging as a major problem in Kashmir. The first case of AE in India was reported in 1980. The patient was a resident of Uri, a mountainous region in Western Kashmir [14,15]. From 1980 to 2012, no cases of AE were reported in Kashmir, as in other parts of India. However, from 2012 onward, several cases of AE in Kashmir have been reported [17,53,54,55,69]. The results of our literature search also revealed striking findings. Of the 126 cases of AE recorded in India, 118 (93.7%) were residents of Kashmir Valley. Three out of the eight cases reported from other parts of India were Indian Soldiers who could have been infected during their frequent postings in the mountainous ranges of Kashmir. Could the recent surge of AE in Kashmir be due to increased awareness, better healthcare facilities, and improved diagnosis? Based on our experience over the years, it seems unlikely. We have prospectively followed 229 echinococcosis cases from 1989 to 2005 at SKIMS, Srinagar, and 301 cases from 2005 to March 2019 at DKMC, Srinagar. All cases had CE, and no cases had AE [70]. In contrast, a prospective study on 110 patients with echinococcosis at DKMC from March 2019 to April 2024 identified 12 (10.9%) AE cases. Thus, the emergence of AE is of recent origin and unlikely to be related to increased awareness of the disease or improved diagnosis. We must consider other factors, including (a). recent changes in the population and behavior of the definitive and intermediate hosts, both in the sylvatic and urban cycle, (b). changing ecology and environment, and (c). the impact of urbanization, deforestation, social unrest, and climate change on predator–prey dynamics and disease load.

The red fox is the preferred host for *E. multilocularis* in Asia and Europe [1,4,71,72,73,74,75,76,77]. Kashmir lies between the Great Himalayas in the Northeast and the Pir Panjal range in the Southwest [78,79]. The central part is connected by the short Zabarwan mountain range. The Zabarwan range extends from Ganderbal in the North to Pampore in the South, and Dachigam National Park is located at the foothills of this range [80]. The Kashmir red fox [*Vulpes vulpes griffithii*] is Kashmir’s most widespread wild canid. It is distributed across the Great Himalayas, Pir Panjal, and Zabarwan ranges of mountains, including Dachigam Park [66,81,82,83]. They feed on voles, mice, rabbits, and other vegetable items [11,66]. Thus, the Kashmir red fox population density determines the sylvatic cycle of *E. multilocularis* in Kashmir. Their preferred habitat is a mixed landscape, and animals adapt very well to human presence. Thus, the altitudes of the Himalayas are a potential risk for the spread of *E. multilocularis*. Human exposure possibly occurs from forest visits for vocational or recreational purposes like hunting. Haymaking, collecting firewood from forests, and eating wild fruits are particularly common in Kashmir and are high risks for contracting *E. multilocularis* [84,85,86].

Domestic dogs (*Canis lupus familiaris*) and cats (*Felis catus*) may uncommonly harbor the tapeworm (synanthropic cycle) [87,88]. However, domestic dogs may be the dominant definitive hosts in the peri-domestic cycle in high endemic zones of southern and western China created by deforestation driven by agriculture [71,72,89,90,91]. The population of dogs (*Canis lupus familiaris*) has grown multifold in Kashmir, as the law prohibits killing, poisoning, or rendering useless any animal in the society [92,93,94]. Most of this population comprises stray dogs with few owners. However, tribal populations often have pet dogs to protect their herds and are intimately associated with them. Dogs may be the source of the spread of *E. multilocularis* to this population, and patients from non-hilly parts of the valley may contract an infection from contact with stray dogs [72,90].

Intermediate hosts, the *Arvicolinae*, are a subfamily of rodents that includes a few types of voles, lemmings, muskrats, and deer mice [9]. Voles are the most populous group of rodentia in the Northern Hemisphere, and their *E. multilocularis* infection rates range from 27 to 80%. There are several species of voles in Kashmir, namely the Central Kashmir Vole (*Alticola montosus*), True’s Vole (*Hyperacrius fertilis*), and the Murree Vole (*Hyperacrius wynnei*) [83,95,96,97,98,99]. The rodent population has become quite a serious problem in Kashmir and a threat to apple orchards. Red foxes, among others, prey on voles. This may impact the predator–prey relationship and contribute to the spread of *E. multilocularis*.

A lemming is a small rodent, usually found in or near the Arctic in tundra biomes, and the arctic fox is a strong candidate for being the most influential lemming predator. The muskrat, native to swamps and wetlands, and deer mouse, with nocturnal habits spending most of the daytime in burrows, are small rodents prevalent in North America and are vulnerable to predation by coyotes, foxes, and raccoons and have a high prevalence (≈22.3%) of *E. multilocularis* infection. Some other herbivorous mammals, including lagomorphs (e.g., pika), pigs, boars, horses, cattle, nonhuman primates, the Tibetan hare, and dogs, can become aberrant hosts for the larval stage and contribute to the spread of *E. multilocularis*.

*Echinococcus multilocularis* is distributed across the northern hemisphere. The eggs need optimal environmental conditions, including high humidity on the superficial ground layer and dense vegetation, to maintain longevity and promote transmission [61]. Because of this, infection is restricted to the northern hemisphere’s moderate to cold climate zones. Kashmir has a cold to temperate climate, high humidity, heavy vegetation, and moist soil, ideal for transmitting *E. multilocularis* [78]. Kashmir is bordered by two highly endemic regions of China: Xinjiang to the north and the Tibet Autonomous Region (Xizang) to the east [79]. The Central Asian Republics, another region endemic to *E. multilocularis*, lie northwest of Kashmir and share sociocultural traditions and phytogeographical relationships [100]. Thus, Kashmir’s geographic and climatic conditions are a potential risk for the endemicity of *E. multilocularis* [101].

Recently, significant events have occurred in Kashmir with social, economic, and ecological consequences. Deforestation and urbanization have occurred at an alarming rate [83,102,103]. Some of the major consequences of deforestation and urbanization have been the prominent land use associated with impacts on biodiversity. As a result, the Kashmir red fox, the principal definitive host for *E. multilocularis*, has lost its habitat and shifted into orchards and parks in the foothills for its needs. This has increased fox–human contact, as this animal is well adapted to human presence. The cases of AE reported in our study lived in and around the orchard garden and parks, and a significant population of red foxes is observed in these areas. Next, Kashmir has passed through a protracted period of civil unrest and conflict with significant social and economic consequences [104]. In such a scenario, the population can use local resources, namely high altitudes, forests, and meadows. These sites are potential risk factors for the spread of *E. multilocularis*. Thirdly, global warming, the greenhouse effect, and climate change are interrelated and devastate ecosystems [105]. Many species shift the latitude and altitude of their habitats in response to climate change [106,107]. Small mammals, including the intermediate host of *E. multilocularis*, show maximum adaptability to moving to high altitudes in response to climate change [108]. This can change predator–prey dynamics and may promote disease transmission. AE is posing a serious threat to a subset of the population in Kashmir and may expand to other regions. We need to move in several directions to face this challenge. Potential risk factors for transmission need to be identified. Dedicated studies using mass ultrasonography in high-risk populations are essential to identify the disease load. Molecular studies on human material [mitochondrial gene sequences (cox1, nad2, and cob) and DNA microsatellites] can define the haplotype involved and help trace the organism’s global movement. Animal studies on definitive and intermediate hosts involved in the sylvatic and synanthropic cycles should be conducted to understand predator–prey dynamics. Finally, randomized trials using albendazole with or without praziquantel and guidelines for managing early and advanced disease must be drafted with socioeconomic and local healthcare facilities in mind. Table 4 depicts why Kashmir is an ideal location for the transmission of *E. multilocularis*, the potential high risks of exposure, possible reasons for the disease’s emergence, and proposed future directions for research to face the challenge of the high endemicity of *E. multilocularis* in Kashmir.

The clinical profiles of our patients with AE were distinctive. The liver was the primary site of involvement in all 12 cases [1,109]. The AE tumors grow to large, bulky masses with invasive potential to spread to the hilum and hilar structures, liver capsule, adjacent organs, and distant spread [21]. The tumors in the present study had reached a mean maximal diameter of 9.22 ± 3.21 cm, with tumor volumes of 426 ± 374.61 cm^3^. All showed invasive features and involved hepatic vasculature and biliary tract centrally and adjacent organs peripherally. Early on, smaller tumors are usually asymptomatic and picked up incidentally during routine imaging for unrelated causes [110]. Two cases in the present study had such incidental diagnoses. Patients with larger tumor masses presented with non-specific symptoms, including RUQ, fatigue, and vague aches and pains with minimal abnormalities of liver tests. The advanced stage of the disease presents several syndromes [15,111,112]. Two patients in the present study had tumors metastasizing to the lungs [113]. In advanced stages, patients present with compromised liver mass and liver failure and are uniformly fatal if left untreated [4].

Diagnosis of AE in the present study was made based on findings of imaging tools, serology, and histology [20,21]. Ultrasonography had the advantage of identifying liver lesions, especially in asymptomatic subjects. It was a good imaging tool for determining the size and echogenicity of the mass, necrotic areas, irregular borders, dilatation of bile ducts, and pattern of calcification. Doppler ultrasound identified hepatic vascular structures within and outside the tumor and gave a fair idea of encasement, compression, or involvement of vascular structures [114]. Because of its availability, cost, and non-invasive nature, we used ultrasonography in the follow-up for any interim changes in tumor size and any new findings. However, ultrasonography has limitations in evaluating the tumor structure and extent. Non-contrast computed tomography was an excellent tool for a global view of the tumor mass, calcification within the tumor mass, and its extension within and outside the liver [5]. Contrast-enhanced computed tomography showed no enhancement of the mass. However, few cases showed peripheral contrast enhancement due to perilesional fibroinflammatory tissue. We used magnetic resonance imaging to complement computed tomography in defining the tumor’s internal structure [17,115,116]. T1WI showed a hypo or isointense lesion, while T2WI revealed both hypointense (solid) and hyperintense (cystic) components. T2W hyperintense small, smooth, rounded cysts of less than 1 cm represent metacestode vesicles, whereas T2W hyperintense large, irregular cystic areas reflect liquefaction necrosis. T2W hypointense solid component is formed by coagulation necrosis, granuloma, fibrous tissue, and calcification. The appearances on T2WI have been classified by Kodama et al. into five types (Type 1 to 5), representing the progressive stages of the disease. Diffusion-weighted imaging further helped in tumor characterization, and apparent diffusion coefficient (ADC) values were useful in differentiating AE tumor masses from liver cancer with restricted diffusion and liver cysts with higher free diffusion. The ADC values of AE masses are higher than liver cancer and lower than liver cysts. Magnetic resonance cholangiopancreatography (MRCP) was a non-invasive tool for visualizing the biliary tree and planning interventional treatment or surgery, as the case may be. Evaluation of metabolic activity and viability of parasitic lesions through ^18^F-FDG uptake is an important breakthrough in AE imaging [27,117]. ^18^F-FDG PET-CECT-Scans showed increased uptake in the lesion’s periphery, while the center does not take up ^18^F-FDG. Serodiagnosis of *echinococcosis* performed by ELISA using E. granulosus fluid yields a diagnostic accuracy of 95% for both CE and AE [18,19]. *E. multilocularis-specific* ELISA using Em2 antigens has a high diagnostic sensitivity of 90–100% and a specificity of 95–100% for AE, allowing discrimination between CE and AE [118].

The WHO-IWGE PNM classification based on imaging findings was established to denote the pattern, extension, and invasion of parasitic mass in the liver (P), the involvement of adjacent organs (N), and distant metastases (M) [31,119]. The disease is divided into four stages based on P (PX, P0, P1, P2, P3, and P4), N (NX, N0, N1), and M (MX, M0, M1) findings. PNM classification and disease staging determine the current treatment strategies. Most cases had advanced disease (stage IIIB or IV) and were inoperable.

AE treatment options are determined by disease staging based on PNM classification and availability of healthcare quality [4,5]. Radical liver resections, either lobar or segmental, are curative and indicated for patients with tumors placed in peripheral parts of the liver and without the involvement of major blood vessels or bile ducts [49]. Of the 12 cases in our study, 3 had limited disease and were amenable to segmental or lobar resections. However, the majority of our patients were diagnosed at advanced stages, and radical resections were not possible and were treated with long-term chemotherapy. We used albendazole instead of mebendazole, as it has better bio-availability [33,34]. The drug decreases the recapture of glucose and its union with B-tubulin, which generates metabolic and structural alterations in the parasite. The drug suppresses metacestode growth and stabilizes tumor size. This causes suppression of metacestode growth and tumor regression. Survival improves compared with historical controls (10-year survival: 80–83% versus 0–25%, 15-year survival: 53–83% versus 0%, respectively). In select cases, we combined albendazole with praziquantel, which is known to improve anti-parasitic effectiveness and is well tolerated with mild adverse effects [35]. Liver transplantation can be offered to patients with extensive liver involvement or liver failure [45,120,121]. However, none of our patients could afford liver transplants owing to their poor availability and financial constraints. Instead, 10 of the 20 patients from Central Asian countries had live donor transplants performed in India. Local recurrence or distant metastases occur in around half of patients within one year. Six-year survival of 66% has been reported. Given disease recurrence, adjuvant post-transplant chemotherapy is recommended [122].

We conclude Kashmir is emerging as a high-endemic zone for AE. Twelve cases were registered and treated from March 2019 to April 2024 at DKMC, Srinagar, Kashmir, India. The liver was the primary site of involvement in all cases. Three cases had Stage I disease, amenable to liver resections, and the other nine had advanced disease (Stages IIIB and IV), with tumor invasion of vessels and bile ducts in eight cases and liver capsule and adjacent organs in nine cases. The disease had metastasized to the lung in two cases. A literature review identified 146 cases of AE reported from India from 1980 to April 2024. Of these, 118 (93%) were inhabitants of Kashmir Valley. The disease was prevalent in 22 villages in or near the Himalayas’ foothills.

## Figures and Tables

**Table 1:** Demographic characteristics, clinical features, tumor mass appearances, disease classification, and staging and treatment of 12 cases of alveolar echinococcosis from Kashmir studied at Dr. Khuroo’s Medical Clinic from March 2019 to April 2024.

No.	Age (Year), Gender *	Clinical Disease [Duration]	# of Lesions	Lobeµ [Segments ^¶^]	L × B × H cm ^Ω^ [Volume mL ^¥^]	PNM [Stage] ^€^	Treatment ^£^ [Follow Up, Outcome]
1	58F	Pain RHQ **, Fatigue [8 months]	1	RL [7,6, and 5]	13.0 × 9.0 × 9.0 [978.9]	P4N1M0 [IV]	ALB + PQ [Five-year clinical improvement. No disease progression. MRI and PET CT at a five-year follow-up showed disease activity.]
2	50M	Pain RHQ, Icterus [2 years]	1	RL [5 and 6]	7.0 × 6.0 × 6.0 [170.3]	P1N0M0 [I]	Surgery + ALB [Well at three years and nine months, in follow-up.]
3	50F	Pain RHQ [2 months]	1	LL [2 and 3]	6.5 × 6.0 × 6.0 [158.6]	P1N0M0 [I]	Surgery + ALB [Well at three years and four months, in follow-up.]
4	24M	Pain RHQ [2 years]	1	RL [8 and 5]	9.0 × 8.0 × 5.1 [249.6]	P1N0M0 [I]	Surgery [declined], ALB [Lost to follow-up at one year.]
5	45F	Incidental -	2	RL [8]	3.5 × 3.5 × 3.0 [24.9]	P3N1M0 [IIIB]	ALB [Well at two years and four months, in follow-up.]
				RL [5 and 6]	10.9 × 9.5 × 8.0 [562.9]		
6	40M	Pain RHQ, rever, jaundice [3 years]	1	RL [7, 6, and 5]	16.5 × 14.0 × 4.5 [1376.4]	P4N1M0 [IV]	Aspiration + Surgery + ALB [Residual disease at two years. Well, in follow-up.]
7	43F	Pain RHQ. Aches and pains. [4 years]	1	RL [7 and 6]	8.2 × 7.0 × 6.7 [261.3]	P2N1M0 [IIIB]	ALB [Well at nine months, in follow-up.]
8	40F	Incidental -	2	RL [5 and 6]	10.6 × 10.2 × 7.8 [573.3]	P4N1M0 [IV]	ALB + PQ [Clinical improvement at one year. MRI at one-year follow-up showed disease activity.]
				LL [4, 3, and 2]	12.0 × 10.0 × 7.0 [571.1]		
9	74M	Pain RHQ [6 years]	2	RL [7 and 6]	6.8 × 4.7 × 3.7 [80.3]	P4N1M1 [IV]	ALB + PQ [Clinical improvement at four months.]
				LL [2 and 3]	7.8 × 6.1 × 5.4 [174.2]		
10	45F	Pain RHQ [3 months]	1	RL [7 and 8]	11.0 × 9.8 × 9.2 [674.7]	P2N1M1 [IV]	ALB + PQ [Clinical improvement at three months.]
11	50F	Pain RHQ [2 years]	1	LL/RL [4B and 5]	9.1 × 8.8 × 7.6 [413.8]	P2N1M0 [IIIB]	ALB + PQ [Under evaluation.]
12	45F	Pain RHQ [2 years]	1	RL [7 and 6]	6.5 × 5.4 × 5.4 [120.5]	P1N1M0 [IIIB]	ALB +PQ, [Under evaluation.]

# = number, * M = Male, F = Female, ** RHQ = Right hypochondrium, µRL = right lobe liver, LL = left lobe liver, ^¶^ Segments = as per Couinaud classification, ^Ω^ L × B × H refers to the length, breadth, and height of the tumor mass, ^¥^ Tumor volume was determined using HOROS 3.3.5 Imaging software, ^€^ PNM = WHO-IWGE, World Health Organization Informal Working Group on Echinococcosis classification, ^£^ ALB = albendazole, PQ = praziquantel.

**Table 2:** This table depicts the systematic review results and details of 146 cases of AE in India from 1980 to April 2024.

Author Year	# Cases	Residence	Age (Years) Gender	Liver Disease		Stage (SI–SIV) # Cases	Surgeries	Reference
				Lobe # Cases	Size cm			
Systematic Search A.								
Khuroo et al., 1980	1	Kashmir, India	29 M	RL	14 × 18	SIV	Surgery, died	[15]
Taneja et al., 1990	1	India (region not known)	60 M	Peritoneum and spleen	_	SIV	Splenectomy	[42]
Shaw et al., 2010	1	Indian Soldier	31 M	RL	_	_	Aspiration, excision	[43]
Tyagi et al., 2010	1	India (region not known)	25 M	Brain	_	_	Resection	[44]
Vijay et al., 2013	1	Indian soldier	43 M	RL 6, 5, 7 & 8	7.4 × 6.6 × 7.9	SIV	-	[13]
Jha et al., 2015	1	Kyrgyzstan	31 F	RL, LL	_	SIV	Liver transplantation	[45]
Madhusudhan et al. 2015	1	India (region not known)	45 M	_	_	_	Resection	[46]
Bhatia et al., 2016	1	Indian Soldier	37 M	LL/RL	17	_	Left hepatectomy	[12]
Prabhakar et al., 2017	1	India (region not known)	27 F	RL	14	_	_	[47]
Bansal et al., 2018	3	Kyrgyzstan	_	_	_	_	_	[48]
Goja et al., 2018	4	Central Asian countries	33.7 ± 3.1 M1:F3	Liver	_	SIV 4	Liver transplantation 3, Tri-segmentectomy 1	[49]
Dudha et al., 2018	1	Peruvian islands, Peru	28 F	Lung	45 × 3.7	-	Resection	[50]
Yattoo et al., 2018	10	Kashmir, India	39.0 ± 11.0. M5:F5;	RL 8, LL 2	3.5 to 15	SI 2, SII 4, SIII 3, SIV 1	ERCP 3	[16]
Mir et al., 2019	13	Kashmir, India	42.07 ± 8.88 M5:F8	RL 2, LL 2, RL/LL 9	-	-	Resection 8, aspiration 1, unresectable 4	[51]
Talwar et al., 2020	1	Iraq	7 F	RL/LL	_	SIV	Died two months after follow-up	[52]
Jehangir et al., 2020	6	Kashmir, India	32.8 ± 11.2. M2:F4	RL 4, LL 2	5.9 to 9.6	SII 5, SIV 1	Resection 2	[53]
Parry et al., 2020	23	Kashmir, India	_	Liver	_	SI 7, SI I5, SIII 7, SIV 4	_	[17]
Ahmad et al., 2021	25	Kashmir, India	53.4 (30 to 70). M12:F13	Liver	_	_	_	[54]
Mitra et al., 2024	1	Kashmir, India	36 M	RL	12.5	_	Resection	[55]
Systematic search B.								
Khuroo et al. (present study)	12	Kashmir, India	46.6 ± 11.9. M4:F8	RL 11, LL 3, RL/LL 1	3.5 to 16.5	SI 3, SIIIb 3, SIV 6	Surgery 4, aspiration 1	-
Systematic search C.								
Systematic literature review	38	Kashmir, India 27, West Bengal, India 1, Central Asia 10	_	Liver	_	_	LT6, resection 11.	-

# = number of cases

**Table 3:** Global occurrence of human cases of alveolar echinococcosis.

Country	Region	Total Cases Reported	Cases Per Year	Prevalence (n/10^5^)	Incidence (n/10^5^/year)	Reference
India	Kashmir (22 villages/5 districts)	118 (2012–2024)	9.3	146	10.3	-
China	Seven Counties	230,000 (Population 26.6 × 10^6^)	16,629	960	7.38	[6,64]
	Sichuan	-	2390	3600	-	
	Qinghai	-	3766	1000	-	
	Ningxia	-	17,760	2000	-	
	Gansu	-	7676	2900	-	
	Tibet	-	172	100	-	
	Xinjiang	-	811	200	-	
Russia	Europe to Siberia	-	1180	-	-	[61]
Kyrgyzstan	Osh Oblast, Naryn Oblast	1319 (1996–2016)	17	-	-	[9,57]
	Naryan Oblast (Kochkor district)	-	-	1970	7.1	
	Osh Oblast (Alay district)	-	-	6400	6.0	
Tajikistan	Dushanbe	22 (2010–2013)	20	-		
Kazakhstan	Almaty Oblast, Akmola Oblast	56 (2006–2015)	39	-	-	
Uzbekistan	-		24			
Turkmenistan	-	-	2			
Turkey	SE Antolia, Erzurum province, Izmir, Diyarbakir province,	230	100	-	-	
	SE Anatolia	48	-	0.4	0.63	
Iran	Razavi Khorasan province (1997–2012)	18	11	-		[65]
Japan	Hokkaido	2316 (1937–2016)	12	-	0.013	[59]
EU	Germany, France, Poland, Austria, Belgium, Lithuania, Sweden, Slovakia,	703 (2016–2020)	114	-	0.15–0.21	[60,61]
	The country-wise number of cases recorded from 2016 to 2020 is as follows: Germany (220), France (204), Poland (103), Lithuania (68), Austria (41), Belgium (27), Slovakia (18), and Sweden (12). No cases were recorded in the UK.					
Canada	Alberta	17 (2013–2020)	-	-	0.064	[62,63]

**Table 4:** Alveolar echinococcosis in Kashmir, India. Potential risk factors for transmission and possible causes for the recent emergence of disease.

	Particulars	Comments
Kashmir is an ideal location for the existence of *E. multilocularis.*		
	Himalayan altitudes	Habitat for the definitive host [Kashmir red fox]
	Extensive network of national parks, wildlife sanctuaries, and Ramsar sites	Kashmir red fox visits these for food
	Forests, alpine meadows, prairies, steppes	Habitat for several types of rodents [intermediate hosts]: the Central Kashmir Vole (*Alticola montosus*), the True’s Vole (*Hyperacrius fertilis*), and the Murree Vole (*Hyperacrius wynnei*)
	High humidity on the superficial ground layer, dense vegetation, and cold weather	Optimal environmental conditions for eggs to maintain longevity and promote transmission
	Predator–prey relationship [red fox–rodents].	Maintains sylvatic cycle of *E. multilocularis* in Kashmir
Potential high-risk factors for transmission		
	Living or visiting high altitudes	Tribal populations. High altitudes cause exposure to *E. multilocularis*
	Work or visiting parks and wildlife sanctuaries	Contact with definitive host [fox] leads to exposure
	Dog ownership or contact with stray dogs	Promotes cycle for *E. multilocularis* and leads to human exposure
	Eating wild fruit, chewing grass, or haymaking	Exposure to infection
Possible causes of the emergence of AE in Kashmir		
	Increased awareness, better healthcare facilities, and improved diagnosis	Unlikely [see text for explanation]
	Developing orchards in the foothills of the Himalayas	Frequent visits by red fox for food, causing human exposure
	Menace of rodents in households and fields	Promotes both the sylvatic and synanthropic cycles of disease
	Menace of stray dogs in populated areas	Synanthropic cycle and human exposure
	Deforestation and urbanization	Migration of red foxes to populated areas, leading to human exposure
	Prolonged social unrest and conflict	Use of local resources, namely high altitudes, forests, and meadows, for livelihoods
	Global warming, the greenhouse effect, and climate change	Latitude and altitude shift of small mammals [intermediate hosts] causing changes in predator–prey dynamics.
Proposed future directions for research to face the challenge of the high endemicity of *E. multilocularis* in Kashmir.		
	Define potential risk factors for human transmission	Case–control studies; cohort studies.
	Continued studies on disease load	Mass ultrasonography studies in endemic zones
	Molecular studies on human material [mitochondrial gene sequences (cox1, nad2, and cob) and DNA microsatellites]	Define the haplotype prevalent in Kashmir [Asian, Mongolian, European, and North American]
	Animal studies	Identify definitive and intermediate hosts in sylvatic and urban cycles
	Management	Randomized trials using albendazole with or without praziquantel Guidelines for managing early and advanced disease, keeping socioeconomic and local healthcare facilities in mind

## Data Availability

The data of patients at Dr. Khuroo’s Medical Clinic, presented as a case series, are available upon request from the corresponding author. However, detailed information and data, along with the patient’s identity, cannot be divulged due to privacy and ethical restrictions. Published data on alveolar echinococcosis can be accessed from various databases, as indicated in the Systematic review. Data of patients procured from other medical centers is available with the primary physicians.

## Supplementary Materials

The following supporting information can be downloaded at: https://www.mdpi.com/article/10.3390/life14070794/s1, Document S1 Volumetry and Document S2 Systematic Review.

## Abbreviations

DKMC = Dr. Khuroo’s Medical Clinic; *E. multilocularis* = *Echinococcus multilocularis*; CE = Cystic echinococcosis; AE = Alveolar echinococcosis; DALYs = Disability-adjusted life years; YLL = Years of life lost owing to mortality; YLD = Years of healthy life lost owing to disability; CECT = Contrast-enhanced computed tomography; CE-MRI = Contrast-enhanced magnetic resonance imaging; MRCP = Magnetic resonance cholangiopancreatography; PAS = Periodic acid-Schiff stain; PRISMA = Preferred reporting items for systematic reviews and meta-Analyses; WHO-IWGE PNM = World Health Organization—Informal Working Group on Echinococcosis-proposed PNM classification.
