# Supplementary material for: A Case Series and Literature Review of Alveolar Echinococcosis in Kashmir, India: An Emerging Endemic Zone for Echinococcus multilocularis
Source: Life (Basel). 2024 Jun 24;14(7):794. doi: 10.3390/life14070794 (PMC11277966; doi:10.3390/life14070794)
Supplement: Supplementary file 1 [file life-14-00794-s001.zip › Supplimentary Annexure Document S1 Volumetery.pdf]

SUPPLEMENTARY
ANNEXURE DOCUMENT S 1.
LIVER VOLUMETRY

A case series and literature review of Alveolar echinococcosis in Kashmir, India: An emerging endemic zone for *Echinococcus multilocularis*.

LIVER VOLUMETRY

Purpose: To document the procedure for liver volumetry for the present study.

Background. Imaging-based volumetry has been increasingly utilized in current clinical practice to obtain accurate measurements of the liver and tumor volumes. This is particularly useful before major hepatic resections and living donor liver transplantation in determining the size of the remnant liver. (1, 2) Liver volumes are an accurate method of predicting resection volume and future liver residual volumes. (3)

Requirements: To determine liver volumetry, the following items are needed:

- MacBook laptop, which accepts the Horos software.
- CD-ROM with recorded images in a DICOM format
- HOROS, a free, open-source medical imaging viewer.(4)

Determinants: The following volumes can be determined:

- Tumor volume
- Total liver volume
- Resection volume
- Future residual liver volume

Procedure: The procedure of determining tumor volume is done as follows:

- **Loading:** Insert a CD-ROM with recorded images in DICOM format into a MacBook with HOROS software loaded. The DICOM data will be automatically extracted from the Disc by the HOROS software and made available on the viewer (Fig panel A).

- **Contouring:** The 2-D viewer allows viewing of the images and editing functions with drop-down tools. The tumor outline is manually outlined in the ROI drop-down menu by right-clicking the mouse (Fig panel B). The contouring process is repeated and completed on image slices 3 to 5 mm thick. Once contouring is complete, a "Generate missing ROIs" menu is given to contour the mass completely. Next, all ROIs are renamed under the same name.

- **Computing:** Next, the "Compute volume function" renders a 3-D image of the tumor (Fig panel C) with accurate volume measurements.

- **Other liver volumes:** Other liver volumes that can be determined include total (Fig panel D) and resection volumes. For determining resection volumes, Cantle's line, which extends from the top of the gall bladder to the suprahepatic inferior vena cava along the middle hepatic vein, is taken as the line separating the right lobe from the left lobe. Liver segmentation can be performed after manually placing specific landmarks into 9 segments according to the Couinaud model and into 4 segments, which is important for surgery planning.(6) Future liver residual volume is determined by subtracting resection volume from total liver volume. (2) Liver resection volumes can predict post-hepatectomy liver failure. (5)

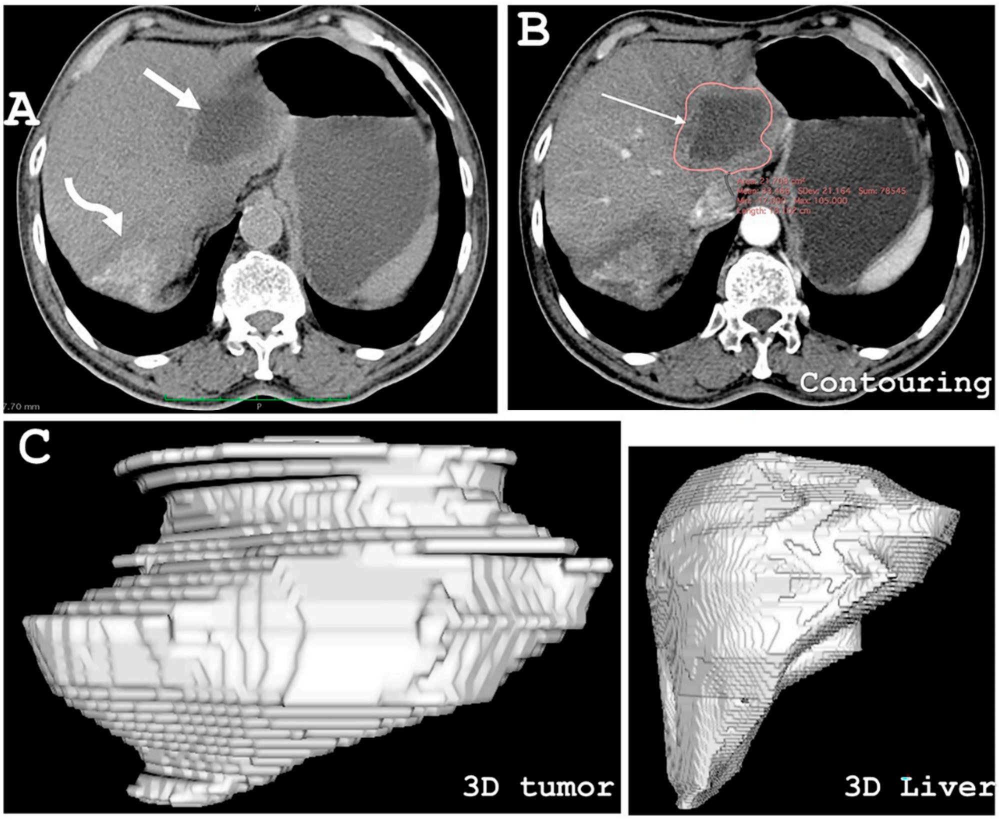

Fig. Liver volumetry. A. Image of the liver extracted from the CD ROM with 2 masses (arrows). B. Contouring the mass in the left lobe with the dimension of the slice. C. 3D image of the tumor mass. D. 3D image of the liver after the liver contouring is done. [Volumes: Left lobe tumor 174.2 cm^3, right lobe tumor 80.3 cm^3. Liver volume 1900 cm^3].

REFERENCES

1. Lima MC, Tana CH, JCaib J, Zhengb J, Kowc AWC. CT volumetry of the liver: Where does it stand in clinical practice? Clinical Radiology journal 2014;y 69 (2014) 887e895.
2. van der Vorst JR, van Dam RM, van Stiphout RS, van den Broek MA, Hollander IH, Kessels AG, et al. Virtual liver resection and volumetric analysis of the future liver remnant using open-source image processing software. World J Surg. 2010;34(10):2426-33.
3. Lodewick TM, Arnoldussen CW, Lahaye MJ, van Mierlo KM, Neumann UP, Beets-Tan RG, et al. Fast and accurate liver volumetry prior to hepatectomy. HPB (Oxford). 2016;18(9):764-72.
4. Elsawaf Y, Anetsberger S, Luzzi S, Elbabaa SK. Three-Dimensional Volumetric Assessment of Resected Gliomas Assisted by Horos Imaging Software: Video Case Series of Postoperative Tumor Analyses. Cureus. 2021;13(2):e13571.
5. Guglielmi A, Ruzzenente A, Conci S, Valdegamberi A, Iacono C. How Much Remnant Is Enough in Liver Resection? Digestive Surgery. 2012;29(1):6-17.
6. Zahel T, Zahel T, Wildgruber M, Roberto, Ardon R, Ardon R. Rapid Assessment of Liver Volumetry by a Novel Automated Segmentation Algorithm. Journal of Computer Assisted Tomography 2013;37(4):577-82.
