# Supplementary material for: A Case Series and Literature Review of Alveolar Echinococcosis in Kashmir, India: An Emerging Endemic Zone for Echinococcus multilocularis
Source: Life (Basel). 2024 Jun 24;14(7):794. doi: 10.3390/life14070794 (PMC11277966; doi:10.3390/life14070794)
Supplement: Supplementary file 1 [file life-14-00794-s001.zip › Supplimentray Annexure Document S2 Systematic review.pdf]

SUPPLEMENTARY
ANNEXURE DOCUMENT S2.
SYSTEMATIC REVIEW

Study: A case series and literature review of Alveolar echinococcosis in Kashmir, India: An emerging endemic zone for Echinococcus multilocularis.

Part A. SYSTEMATIC REVIEW. A systematic review was to document all cases of Alveolar echinococcosis [AE] in Kashmir, India, from 1980 until April 2024.

Protocol. The protocol was established under PRISMA (Preferred Reporting for Systematic Reviews and Meta-analysis), which included established guidelines. (1, 2)The PRISMA Statement consists of a 27-item checklist and a four-phase flow diagram. It aims to help authors improve the reporting of systematic reviews and meta-analyses. We have reported on several systematic reviews and meta-analyses under PRISMA guidelines. (3, 4)Fig 1 shows the flow diagram of the systematic review.

Selection criteria: A patient was accepted to have AE based on clinical, serological, imaging, and histological proof of Echinococcus multilocularis.

Search methods. To find out the cases of AE, we performed three search methods:

- Literature Research [Published cases of AE];
- Casas of AE at DKMC.(Dr. Khuroo's Medical Clinic, Srinagar, Kashmir, India).
- Cases of AE retrieved from other medical Centers.

Literature Research [Published cases of AE].

Primary search: On April 10, 2024, we conducted a primary literature search from PubMed for all published articles using the following MeSH terms: Alveolar echinococcosis and India, Echinococcosis multilocularis and India, and Alveolar hydatid and India.
We found the following articles published in PubMed under each heading:
Alveolar echinococcosis and India: 19 articles [Table 1A].
Echinococcosis multilocularis and India: 23 articles [Table 2A].
Alveolar hydatid and India: 32 articles [Table 3A].

Expanded search: The search was expanded through several areas: -

- other search engines (EMBASE and Google Scholar),
- Conference abstracts from various scientific societies from India (Indian Society of Gastroenterology, Indian National Association for Study of Liver, and Asian Pacific Association for Study of Liver),
- Dissertations and Thesis of Kashmir University and SKIMS Deemed University,
- Personal contacts of GI and surgical clinical units, liver transplant centers, imaging units, and pathology departments.

This search yielded five more articles fulfilling the selection criteria—table 4A.

	Literature Search yielded a total of 79 articles.
	**Duplication**: Duplication was checked by comparing authorship, titles, journal, and year of publication of all articles in the search engine of Endnote 21 and confirmed manually. 35 duplicates were found. 35 duplicates were removed, yielding 44 articles. See Table 5A.
	**Selection Criteria from Published Articles**. Two authors (MSK and NSK) independently reviewed 44 articles for selection criteria. We selected articles from India that published case reports, case series, and case studies of patients with AE. Any discrepancies were resolved by consensus. Based on this, 25 articles were excluded (22 included no case of AE and three published cases of AE from other countries). 19 articles fulfilled the selection criteria for the systematic review (Table 6A).
	**Data synthesis**. We tabulated information from 19 published studies (Table 7A). It included the author, reporting department, study period, study protocol, number of cases of AE reported, residence of patients studied, age and gender, site of liver involvement, serology, stage of disease, and treatment, including liver transplant and surgery. Based on this, 96 cases of AE were identified.
	**Inclusion criteria**: Two authors (MSK and NSK) independently had a full-text review of all articles and studied cases published to confirm the inclusion criteria of AE. Any discrepancies were sorted out by consensus. All cases published fulfilled the inclusion criteria of AE.
	**Demographic data:** We recorded the nationality of all cases. For Indian patients, we recorded residences from Kashmir and other parts of India. Ten cases were foreigners, and 86 were Indians. Out of 86 Indian patients, 79 were residents of Kashmir, while 7 belonged to other parts of India.
**Casas of AE at DKMC (Dr. Khuroo's Medical Clinic).**	
	Search: All cases registered and treated at DKMC from March 2019 to April 2024 were listed. 12 cases of AE were treated at DKMC.
	Duplication: Based on inquiries and records, duplications of such patients to other centers were sought. There were no duplications.
	Inclusion criteria: Two authors (MSK and NSK) independently reviewed case records of all cases to confirm inclusion criteria. All cases were confirmed to fulfill inclusion criteria,
	**Demographic data:** The nationality of all cases was recorded. For Indian patients, we recorded residences from Kashmir and other parts of India. All patients were permanent residents of Kashmir Valley.
**Cases retrieved from other medical Centers:** Retrieval of unpublished cases of AE registered and treated in other medical centers of Kashmir and North India from March 2019 to April 2024	
	We approached various clinical units, liver transplant centers, imaging units, pathology departments from Kashmir, and several tertiary care hospitals from North India. A request was made to give us data on cases of AE registered and managed under their care for the above-specified period, but these have not been

<table>
<tr><td></td><td>published. We received a positive response from several units and a list of 39 patients with AE, along with their identity, contact details, residence, clinical, serological, imaging details, and histological findings.</td></tr>
<tr><td></td><td>Duplication: This list was analyzed for duplications.
One case was registered in 2 centers, giving a final list of 38 cases, and excluded.</td></tr>
<tr><td></td><td>Inclusion criteria: Two authors (MSK and NSK) independently reviewed all case records to confirm inclusion criteria. In case of incomplete medical records, the treating unit or patient was contacted for full medical records. All cases were confirmed to fulfill inclusion criteria.</td></tr>
<tr><td></td><td>Demographic data: We recorded the nationality of all cases. For Indian patients, we recorded residences from Kashmir and other parts of India. Ten cases were foreigners, and 28 were Indians. Out of 28 Indian patients, 27 were residents of Kashmir, while one belonged to other parts of India.</td></tr>
<tr><td colspan="2">The cases identified from all three search methods were listed together. This yielded 146 cases of AE, 20 foreigners, 118 from Kashmir, and 8 from other parts of India.</td></tr>
<tr><td colspan="2">The detailed information of 146 cases of AE from India is as under:</td></tr>
<tr><td colspan="2">Total cases: Information on 146 cases of AE was collected. This included 96 cases published in the literature, 12 cases registered and treated at Dr. Khuroo’s Medical Clinic, and 38 registered and currently managed at various medical Centers in Kashmir and North India.

Residence and Nationality: Of the 146 cases, 20 patients were foreign nationals (Central Asia 18, Iraq 1, and Peru 1). 126 cases were Indian nationals. Of the 126 patients, 118 were residents of Kashmir Valley, and 8 patients were from other parts of India. Of the eight patients from other parts of India, three were Indian Soldiers who possibly were posted in Kashmir and could have contracted the disease from Kashmir.

Age and Gender: Of the 146 cases, one was a child 7 yr. Of age, and all others were adults. There were 64 males and 82 females.

Primary organ: 143 patients had the liver as the primary disease site. Of the remaining three cases, one each had primary disease in the lung, Brain, and spleen/peritoneum.

Disease stage: Stage IV disease was predominant in most cases.

Liver transplant: 14 patients, all foreign nationals, underwent live donor-related liver transplants.

Surgery: 34 patients underwent various types of liver resections and reconstructions of hepatic vessels and bile ducts.

Deaths: Four deaths were reported. Three patients died of disease recurrence after incomplete surgical procedures, and one child with advanced lung disease.</td></tr>
</table>

Residential location of 39 cases (12 cases from Dr. Khuroo's Medical Clinic and 27 registered in various medical centers) of A. echinococcosis from Kashmir Vally. Refer to Fig 2 which defines the locations pictorially.

Serial number	Region	# cases	Rural / Urban	# villages where cases were reported	Population of villages	Tribal population
1	Ganderbal	10	Rural	6 villages	18,399 (M 10070, F 8329)	Pashtuns, Pathans and Afghans
2	Shopian, Kulgam, and Pulwama	15	Rural	7 villages	24, 565 (13091, F 12795)	-
3	Gurez Valley	5	Rural	3 villages	5,647 (M 2396, F 1930)	Dards, Shins
4	Uri Kupwara	6	Rural	4 villages	15,391 (M 7928, F 7463)	Gujjars
5	Budgam	3	Rural	3 villages	15,195 (M 8330, F 6865)	-
Total	-	39		22 villages	79,197 (M 41815, F37382)	

Based on this, we found that patients came from 22 villages from five border districts of Valley, with a population of 79, 197. No case of AE was reported outside these 22 villages from 5 regions of Kashmir. None of the patients belonged to urban central parts of the Kashmir Valley.

The prevalence and incidence of AE in the affected population were calculated.

Incidence and Prevalence of AE in Kashmir, India. The total number of cases reported from Kashmir was 118 (M 65, F 83) over a period of 12 years (March 2012 to April 2024), originating from a select population of 79,197 (M 41,815, F 37,382) and two reported deaths.

Incident cases per year were 9.83, with and incidence 12.41/105/yr. (Males 11.16/105/yr. and Females 13.81/105/yr.) and the disease prevalence in the affected population was 146.47/105 (Males 131.53/105 and Females 163.18/105)

Purpose of the systematic review: To define cases of alveolar echinococcosis (AE) in Kashmir, India
Inclusion criteria: Cases with clinical, serological, imaging and histological findings of AE.

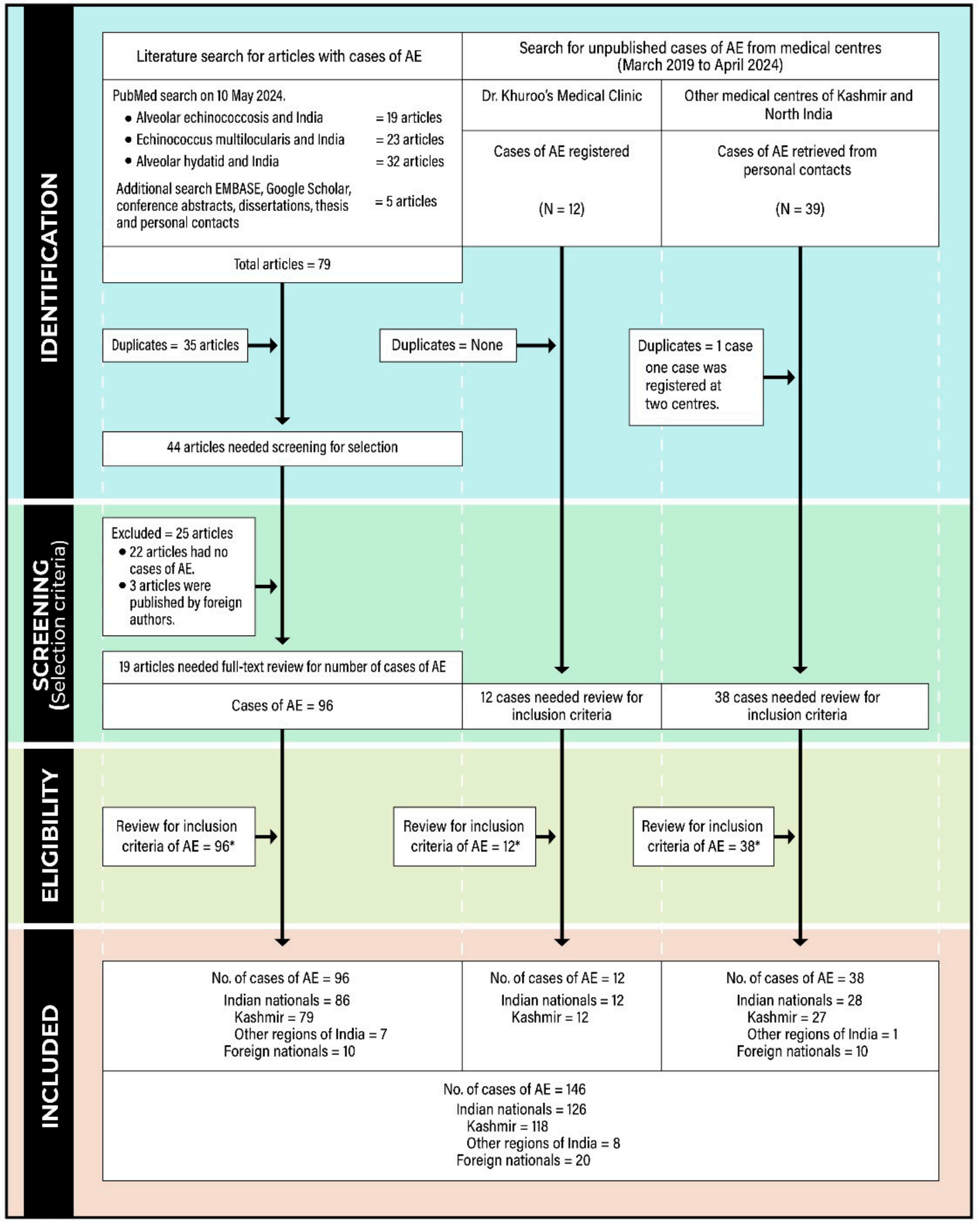

* The inclusion criteria for AE were reviewed independently by two authors (M.S.K. and N.S.K.), and any discrepencies were sorted out by consensus.

Fig1. Flow diagram of systematic review for the study.

Gurez valley:
5 cases of AE,
3 villages.
Population: 5647 (M: 2396, F: 1930).

Gurez Valley lies high within Great Himalayan Range.
Elevation: 2400 m.

Uri / Kupwara:
6 cases of AE.
4 villages.
Population: 15,391 (M: 7928, F: 7463).

Uri is situated high in Pir Panjal Range.
Elevation: 2634 m.

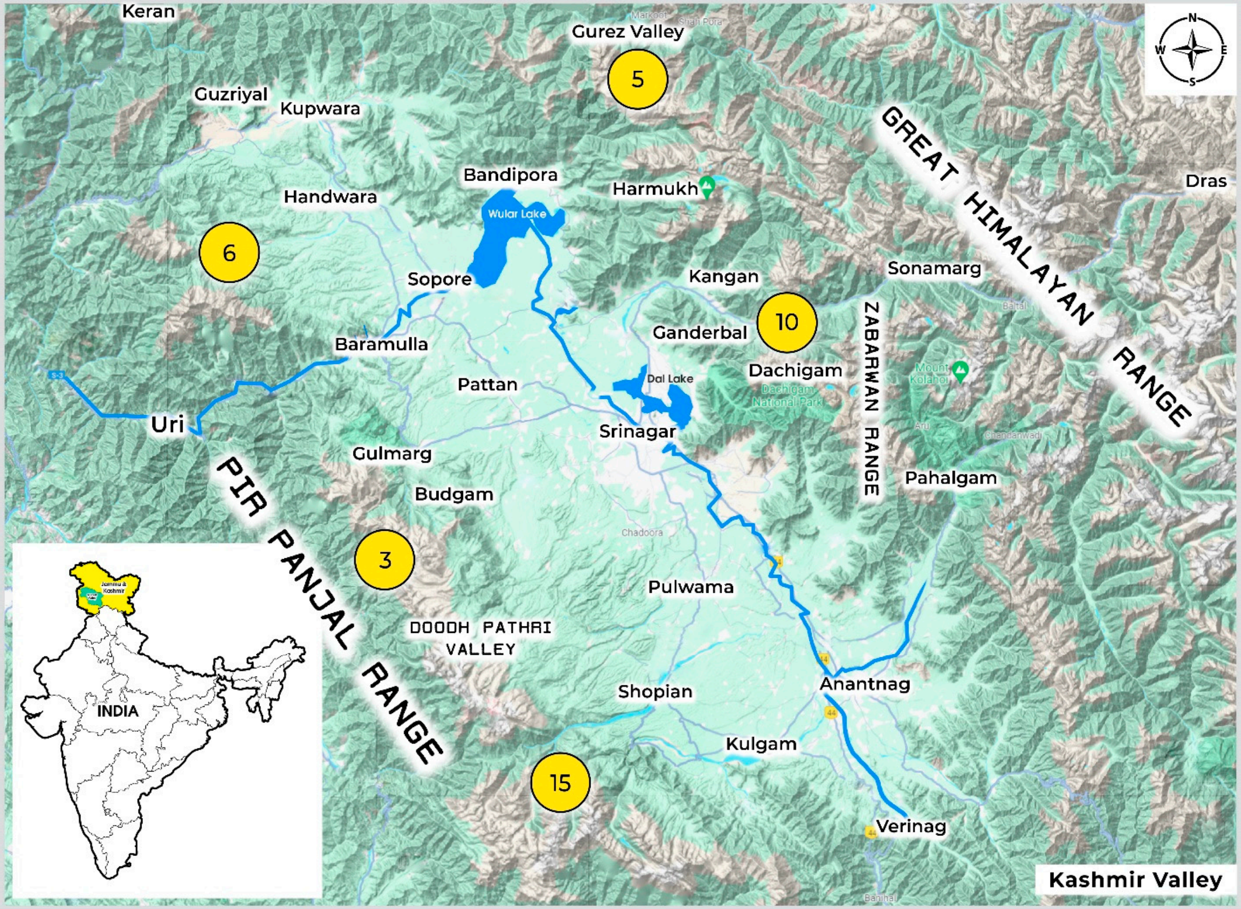

Budgam:
3 cases of AE.
3 villages.
Population: 15,195 (M: 8330, F: 6865).

Budgam lies within Pir Panjal Range and Doodhpathri Valley.
Elevation: 2730 m.

Ganderbal district:
10 cases of AE.
6 villages.
Population: 18,399 (M: 10,070, F: 8329).

These villages are in the foothills of Zabarwan Range and Dachigam National Park.
Elevation: 3966 m.

Shopian / Kulgam / Pulwama:
15 cases of AE,
7 villages.
Population: 24,565 (M: 13,091, F: 12,795).

Shopian is adjacent to Pir Panjal Range.
Elevation: 2057 m.

Fig 2. Residential location of 39 cases of AE from Kashmir Valley.

Table 1A. Systematic review. MeSH term: Alveolar echinococcosis and India. Search Engine: PubMed. Date: 10th May 2024. No of articles retrieved: 19.

1: Dietrich CF, Douira-Khomsi W, Gharbi H, Sharma M, Cui XW, Sparchez Z, Richter J, Kabaalioğlu A, Atkinson NSS, Schreiber-Dietrich D, Yi D. Cystic and alveolar echinococcosis of the hepatobiliary tract - the role of new imaging techniques for improved diagnosis. Med Ultrason. 2020 Mar 1;22(1):75-84. doi: 10.11152/mu-2421. PMID: 32096792.

2: Mitra S, Charaya P, Deshpande SG, Parkhi M, Yadav TD. Hepatic alveolar echinococcosis simulating metastatic malignancy. Autopsy Case Rep. 2024 Feb 8;14:e2024474. Doi: 10.4322/acr.2024.474. PMID: 38476731; PMCID: PMC10927241.

3: Parry AH, Wani AH, Feroz I. The spectrum of multimodality imaging findings in hepatic alveolar echinococcosis and the potential role of diffusion-weighted imaging in its characterization. Pol J Radiol. 2020 Nov 10;85:e613-e623. doi: 10.5114/pjr.2020.101015. PMID: 33376563; PMCID: PMC7757515.

4: Madhusudhan KS, Srivastava DN, Dash NR, Venuthruimilli A, Sharma R, Gamanagatti S, Gupta AK. Alveolar Echinococcosis of the Liver: A Diagnostic Problem in a Nonendemic Area. Curr Probl Diag Radiol. 2016 Jan-Feb;45(1):80-3. Doi: 10.1067/j.cpradiol.2014.09.001. Epub 2014 Nov 6. PMID: 25482390.

5: Dudha M, Shaikh Z, Bhaiyat M, Wadiwala IJ, Bhaiyat ZT. A Case of Echinococcal Cyst of the Lung. Respir Med Case Rep. 2018 Oct 10;25:286-292. Doi: 10.1016/j.rmcr.2018.10.007. PMID: 30364676; PMCID: PMC6197673.

6: Madhusudhan KS, Srivastava DN, Dash NR, Venuthurimilli A, Sharma R, Gamanagatti S, Gupta AK. Alveolar echinococcosis of liver: a diagnostic problem in a nonendemic area. Curr Probl Diagn Radiol. 2015 Mar-Apr;44(2):221-6. Doi: 10.1067/j.cpradiol.2014.08.006. Epub 2014 Nov 18. PMID: 25795033.

7: Jha B, Lipi L, Gajendra S, Sharma R, Sachdev R. Alveolar echinococcosis with portal vein thrombosis: An unusual cause for liver transplantation. Asian Pac J Trop Med. 2015 Aug;8(8):683-4 10.1016/j.apjtm.2015.07.014. Epub 2015 Jul 22. PMID: 26321526.

8: Wang J, Cai B, You C. Surgical treatment options for cerebral alveolar echinococcosis: experience in six patients. Neurol India. 2009 Mar- Apr;57(2):157-61. doi: 10.4103/0028-3886.51284. PMID: 19439845.

9: Goja S, Saha SK, Yadav SK, Tiwari A, Soin AS. Surgical approaches to hepatic hydatidosis ranging from partial cystectomy to liver transplantation. Ann Hepatobiliary Pancreat Surg. 2018 Aug;22(3):208-215. doi: 10.14701/ahbps.2018.22.3.208. Epub 2018 Aug 31. PMID: 30215042; PMCID: PMC6125266.

10: Kinkar L, Laurimäe T, Acosta-Jamett G, Andresiuk V, Balkaya I, Casulli A, Gasser RB, González LM, Haag KL, Zait H, Irshadullah M, Jabbar A, Jenkins DJ, Manfredi MT, Mirhendi H, M'rad S, Rostam Nejad M, Oudni-M'rad M, Pierangeli NB, Ponce-Gordo F, Rehbein S, Sharbatkhori M, Kia EB, Simsek S, Soriano SV, Sprong H, Šnábel V, Umhang G, Varcasia A, Saarma U. Distinguishing Echinococcus granulosus sensu stricto genotypes G1 and G3 with confidence: A practical guide. Infect Genet Evol. 2018 Oct;64:178-184. doi: 10.1016/j.meegid.2018.06.026. Epub 2018 Jun 21. PMID: 29936039.

11: Khan A, Ahmed H, Simsek S, Liu H, Yin J, Wang Y, Shen Y, Cao J. Molecular characterization of human Echinococcus isolates and the first report of E. canadensis (G6/G7) and E. multilocularis from the Punjab Province of Pakistan using sequence analysis. BMC Infect Dis. 2020 Apr 3;20(1):262. doi: 10.1186/s12879-020-04989-6. PMID: 32245373; PMCID: PMC7118937.

12: Kinkar L, Laurimäe T, Acosta-Jamett G, Andresiuk V, Balkaya I, Casulli A, Gasser RB, van der Giessen J, González LM, Haag KL, Zait H, Irshadullah M, Jabbar A, Jenkins DJ, Kia EB, Manfredi MT, Mirhendi H, M'rad S, Rostami-Nejad M, Oudni-M'rad M, Pierangeli NB, Ponce-Gordo F, Rehbein S, Sharbatkhori M, Simsek S, Soriano SV, Sprong H, Šnábel V, Umhang G, Varcasia A, Saarma U. Globalphylogeography and genetic diversity of the zoonotic tapeworm Echinococcus granulosus sensu stricto genotype G1. Int J Parasitol. 2018Aug;48(9-10):729-742. doi: 10.1016/j.ijpara.2018.03.006. Epu2018 May 19. PMID:29782829.

13: Korhonen PK, Kinkar L, Young ND, Cai H, Lightowlers MW, Gauci C, Jabbar A, Chang BCH, Wang T, Hofmann A, Koehler AV, Li J, Li J, Wang D, Yin J, Yang H, Jenkins DJ, Saarma U, Laurimäe T Rostami-Nejad M, Irshadullah M, Mirhendi H, Sharbatkhori M, Ponce-Gordo F, Simsek S, Casulli A, Zait H, Atoyan H, de la Rue ML, Romig T, Wassermann M, Aghayan SA, Gevorgyan H, Yang B, Gasser RB.
Chromosome-scale Echinococcus granulosus (genotype G1) genome reveals the Eg95 gene family and conservation of the EG95-vaccine molecule. Commun Biol. 2022 Mar 3;5(1):199. doi: 10.1038/s42003-022-03125-1. PMID: 35241789; PMCID: PMC8894454.

14: Kinkar L, Laurimäe T, Balkaya I, Casulli A, Zait H, Irshadullah M,Sharbatkhori M, Mirhendi H, Rostami-Nejad M, Ponce-Gordo F, Rehbein S, Kia EB,Simsek S, Šnábel V, Umhang G, Varcasia A, Saarma U. Genetic diversity and phylogeography of the elusive, but epidemiologically important Echinococcus granulosus sensu stricto genotype G3. Parasitology. 2018 Oct;145(12):1613-1622.doi: 10.1017/S0031182018000549. Epub 2018 Apr 17. PMID: 29661261.

15: Bansal N, Vij V, Rastogi M, Wadhawan M, Kumar A. A report on three patients with Echinococcus multilocularis: Lessons learned. Indian J Gastroenterol. 2018 Jul;37(4):353-358. doi: 10.1007/s12664-018-0860-y. Epub 2018 Aug 18. PMID:30121887.

16: Tyagi DK, Balasubramaniam S, Sawant HV. Primary calcified hydatid cyst of the brain. J Neurosci Rural Pract. 2010 Jul;1(2):115-7. doi: 10.4103/0976-3147.71729. PMID: 21808518; PMCID: PMC3139339.

17: Laurimäe T, Kinkar L, Moks E, Romig T, Omer RA, Casulli A, Umhang G, Bagrade G, Irshadullah M, Sharbatkhori M, Mirhendi H, Ponce-Gordo F, Soriano SV, Varcasia A, Rostami-Nejad M, Andresiuk V, Saarma U. Molecular phylogeny based on six nuclear genes suggests that Echinococcus granulosus sensu lato genotypes G6/G7 and G8/G10 can be regarded as two distinct species. Parasitology. 2018 Dec;145(14):1929-1937. doi: 10.1017/S0031182018000719. Epub 2018 May 21. PMID: 29781421.

18: Bajpai J, Jain A, Kar A, Kant S, Bajaj DK. "Necklace in the lung:" Multilocularis hydatid cyst mimicking left-sided massive pleural effusion. LungIndia. 2019 Nov-Dec;36(6):550-552. doi: 10.4103/lungindia.lungindia_76_19. PMID: 31670306; PMCID: PMC6852218.

19: Singh S, Khichy S, Singh M, Gill JS. Recurrent solitary hydatid cyst of the subcutaneous tissue. Indian J Surg. 2009 Jun;71(3):162-4. doi: 10.1007/s12262-009-0044-1. Epub 2009 Jun 10. PMID: 23133143 PMCID: PMC3452473.

Table 2A. Systematic review. MeSH term: Echinococcus multilocularis and India. Search Engine: PubMed. Date: 10th May 2024. No of articles retrieved: 23
1: Khan A, Ahmed H, Simsek S, Liu H, Yin J, Wang Y, Shen Y, Cao J. Molecular characterization of human Echinococcus isolates and the first report of E. canadensis (G6/G7) and E. multilocularis from the Punjab Province of Pakistan using sequence analysis. BMC Infect Dis. 2020 Apr 3;20(1):262. doi: 10.1186/s12879-020-04989-6. PMID: 32245373; PMCID: PMC7118937.
2: Bhalla VP, Paul S, Klar E. Hydatid Disease of the Liver. Visc Med. 2023 Oct;39(5):112-120. doi: 10.1159/000533807. Ep 2023 Sep 25. PMID: 37899792; PMCID: PMC10601525.
3: Kowalczyk M, Kurpiewski W, Zieliński E, Zadrożny D, Klepacki Ł, Juśkiewicz W, Lasocki J, Dyśko Ł, Batia K, Pesta W. A rare case of the simultaneous location of Echinococcus multilocularis in the liver and the head of the pancreas: case report analysis and review of literature. BMC Infect Dis. 2019 Jul 24;19(1):661. doi: 10.1186/s12879-019-4274-y. PMID: 31340769; PMCID: PMC6657101.
4: Srinivas MR, Deepashri B, Lakshmeesha MT. Imaging Spectrum of Hydatid Disease: Usual and Unusual Locations. Pol Radiol. 2016 Apr 26;81:190-205. doi: 10.12659/PJR.895649. PMID: 27231490; PMCID: PMC4868106.
5: Korhonen PK, Kinkar L, Young ND, Cai H, Lightowlers MW, Gauci C, Jabbar A, Chang BCH, Wang T, Hofmann A, Koehler AV, Li J, Li J, Wang D, Yin J, Yang H, Jenkins DJ, Saarma U, Laurimäe T, Rostami-Nejad M, Irshadullah M, Mirhendi H, Sharbatkhori M, Ponce-Gordo F, Simsek S, Casulli A, Zait H, Atoyan H, de la Rue ML, Romig T, Wassermann M, Aghayan SA, Gevorgyan H, Yang B, Gasser RB. Chromosome-scale Echinococcus granulosus (genotype G1) genome reveals the Eg95 gene family and conservation of the EG95-vaccine molecule. Commun Biol. 2022 Mar 3;5(1):199. doi: 10.1038/s42003-022-03125-1. PMID: 35241789; PMCID: PMC8894454.
6: Bansal N, Vij V, Rastogi M, Wadhawan M, Kumar A. A report on three patients with Echinococcus multilocularis: Lesson learned. Indian J Gastroenterol. 2018 Jul;37(4):353-358. doi: 10.1007/s12664-018-0860-y. Epub 2018 Aug 18. PMID:30121887.
7: Dudha M, Shaikh Z, Bhaiyat M, Wadiwala IJ, Bhaiyat ZT. A Case of Echinococcal Cyst of the Lung. Respir Med Case Rep. 2018 Oct 10;25:286-292. doi: 10.1016/j.rmcr.2018.10.007. PMID: 30364676; PMCID: PMC6197673.
8: Mitra S, Charaya P, Deshpande SG, Parkhi M, Yadav TD. Hepatic alveolar echinococcosis simulating metastatic malignancy. Autops Case Rep. 2024 Feb 8;14:e2024474. doi: 10.4322/acr.2024.474. PMID: 38476731; PMCID: PMC10927241.
9: Talwar N, Agarwal N, Chugh K. A Unique Case of Cardiac Echinococcus multilocularis. Indian Pediatr. 2020 Dec 15;57(12):1181-1182. doi: 10.1007/s13312-020-2076-3. PMID: 33318327; PMCID: PMC7781830.
10: Kumar K, Zaidi A, Husain N. Ovarian hydatid cyst: an uncommon site of presentation. Autops Case Rep. 2023 Dec 15;13:e2023461. doi: 10.4322/acr.2023.461. PMID: 38149072; PMCID: PMC10750830.
11: Matossian RM, Rickard MD, Smyth JD. Hydatidosis: a global problem of increasing importance. Bull World Health Organ. 1977;55(4):499-507. PMID: 74294; PMCID: PMC2366677.

12: Kanojia RP, Bawa M. Thoracoscopic Transdiaphragmatic Excision of Hepatic Hydatid in Patients with Synchronous Pulmonary Hydatid: A Novel Operative Approach. J Laparoendosc Adv Surg Tech A. 2020 Sep;30(9):1036-1039. doi: 10.1089/lap.2020.0228. Epub 2020 Jul 7. PMID: 32634339.

13: Datta P, Sharma B, Peters NJ, Khurana S, Sehgal R. Bilateral Pulmonary Hydatid Cyst in a Young Child: A Rare Case Report from North India. J Lab Physicians. 2022 Feb 9;14(3):348-350. doi: 10.1055/s-0042-1742420. PMID: 36119419; PMCID: PMC9473927.

14: Aikat BK, Bhusnurmath SR, Cadersa M, Chhuttani PN, Mitra SK. Echinococcus multilocoularis infection in India: First case report proved at autopsy. Trans R Soc Trop Med Hyg. 1978;72(6):619-21. doi: 10.1016/0035-9203(78)90015-9. PMID: 734718.

15: Prabhakar N, Kalra N, Behera A, Das A, Lal A, Dhiman RK, Khandelwal N. Large Heterogeneous Calcified Masses in Liver: A Diagnostic Dilemma. J Clin Exp Hepatol. 2017 Dec;7(4):385-386. doi: 10.1016/j.jceh.2017.09.001. Epub 2017 Oct 3. PMID: 29234206; PMCID: PMC5719463.

16: Bajpai J, Jain A, Kar A, Kant S, Bajaj DK. "Necklace in the lung:" Multilocularis hydatid cyst mimicking left-sided massive pleural effusion. Lung India. 2019 Nov-Dec;36(6):550-552. doi: 10.4103/lungindia.lungindia_76_19. PMID: 31670306; PMCID: PMC6852218.

17: Mohanty MC, Ravindran B. Deficiency of antibody responses to T-independent antigens in gerbils---Meriones unguiculatus. Dev Comp Immunol. 2002 May;26(4):385-91. doi: 10.1016/s0145-305x(01)00086-6. PMID: 11888652.

18: Singh S, Khichy S, Singh M, Gill JS. Recurrent solitary hydatid cyst of the subcutaneous tissue. Indian J Surg. 2009 Jun;71(3):162-4. doi: 10.1007/s12262-009-0044-1. Epub 2009 Jun 10. PMID: 23133143; PMCID: PMC3452473.

19: Kushwaha JK, Sonkar AA, Verma AK, Pandey SK. Primary disseminated extrahepatic abdominal hydatid cyst: a rare disease. BMJ Case Rep. 2012 May 30;2012:bcr0220125808. doi: 10.1136/bcr.02.2012.5808. PMID: 22669859; PMCID: PMC4543121.

20: Gandhiraman K, Balakrishnan R, Ramamoorthy R, Rajeshwari R. Primary Peritoneal Hydatid Cyst Presenting as Ovarian Cyst Torsion: A Rare Case Report. J Clin Diagn Res. 2015 Aug;9(8):QD07-8. doi: 10.7860/JCDR/2015/14324.6397. Epub 2015 Aug 1. PMID: 26436004; PMCID: PMC4576597.

21: Khuroo MS, Datta DV, Khoshy A, Mitra SK, Chhuttani PN. Alveolar hydatid disease of the liver with Budd-Chiari syndrome. Postgrad Med J. 1980 Mar;56(653):197-201. doi: 10.1136/pgmj.56.653.197. PMID: 7393812; PMCID: PMC2425836.

22: Taneja K, Gothi R, Kumar K, Jain S, Mani RK. Peritoneal Echinococcus multilocularis infection: CT appearance. J Comput Assist Tomogr. 1990 May-Jun;14(3):493-4. PMID: 2335629.

23: Shaw AK, Gambhir RP, Chaudhry R, Jaiswal SS. Echinococcus multilocularis causing alveolar hydatid disease liver: a rare occurrence in the Indian subcontinent. Trop Gastroenterol. 2010 Apr-Jun;31(2):119-20. PMID: 20862989.

Table 3A. Systematic review. MeSH term: Alveolar hydatid cyst and India. Search Engine: PubMed. Date: 10th May 2024. No of articles retrieved: 32.

1: Srinivas MR, Deepashri B, Lakshmeesha MT. Imaging Spectrum of Hydatid Disease: Usual and Unusual Locations. Pol J Radiol. 2016 Apr 26;81:190-205. doi: 10.12659/PJR.895649. PMID: 27231490; PMCID: PMC4868106.

2: Bhalla VP, Paul S, Klar E. Hydatid Disease of the Liver. Visc Med. 2023 Oct;39(5):112-120. doi: 10.1159/000533807. Epub 2023 Sep 25. PMID: 37899792; PMCID: PMC10601525.

3: Dietrich CF, Douira-Khomsi W, Gharbi H, Sharma M, Cui XW, Sparchez Z, Richter J, Kabaalioğlu A, Atkinson NSS, Schreiber-Dietrich D, Yi D. Cystic and alveolar echinococcosis of the hepatobiliary tract - the role of new imaging techniques for improved diagnosis. Med Ultrason. 2020 Mar 1;22(1):75-84. doi: 10.11152/mu-2421. PMID: 32096792.

4: Bhatia JK, Ravikumar R, Naidu CS, Sethumadhavan T. Alveolar hydatid disease of the liver: A rare entity in India. Med J Armed Forces India. 2016 Dec;72(Suppl 1):S126-S129. doi: 10.1016/j.mjafi.2015.12.005. Epub 2016 Feb 23. PMID: 28050091; PMCID: PMC5192168.

5: Chaudhari V, Sharma AK, Singh BB, Randhawa CS, Uppal SK. Stridor and emphysema due to cystic echinococcosis in cattle and buffalo intermediate hosts in Punjab, India. Vet Parasitol Reg Stud Reports. 2017 Dec;10:51-53. Doi 10.1016/j.vprsr.2017.08.001. Epub 2017 Aug 9. PMID: 31014598.

6: Parry AH, Wani AH, Feroz I. The spectrum of multimodality imaging findings in hepatic alveolar echinococcosis and th potential role of diffusion-weighted imaging in its characterisation. Pol J Radiol. 2020 Nov 10;85:e613-e623. doi: 10.5114/pjr.2020.101015. PMID: 33376563; PMCID: PMC7757515.

7: Mitra S, Charaya P, Deshpande SG, Parkhi M, Yadav TD. Hepatic alveolar echinococcosis simulating metastatic malignancy. Autops Case Rep. 2024 Feb 8;14:e2024474. doi: 10.4322/acr.2024.474. PMID: 38476731; PMCID: PMC10927241.

8: Dudha M, Shaikh Z, Bhaiyat M, Wadiwala IJ, Bhaiyat ZT. A Case of Echinococcal Cyst of the Lung. Respir Med Case Rep. 2018 Oct 10;25:286-292. doi: 10.1016/j.rmcr.2018.10.007. PMID: 30364676; PMCID: PMC6197673.

9: Datta P, Sharma B, Peters NJ, Khurana S, Sehgal R. Bilateral Pulmonary Hydatid Cyst in a Young Child: A Rare Case Report from North India. J Lab Physicians. 2022 Feb 9;14(3):348-350. doi: 10.1055/s-0042-1742420. PMID: 36119419; PMCID: PMC9473927.

10: Kumar K, Zaidi A, Husain N. Ovarian hydatid cyst: an uncommon site of presentation. Autops Case Rep. 2023 De 15;13:e2023461. doi: 10.4322/acr.2023.461. PMID: 38149072; PMCID: PMC10750830.

11: Kanojia RP, Bawa M. Thoracoscopic Transdiaphragmatic Excision of Hepatic Hydatid in Patients with Synchronou Pulmonary Hydatid: A Novel Operative Approach. J Laparoendosc Adv Surg Tech A. 2020 Sep;30(9):1036-1039. doi: 10.1089/lap.2020.0228. Epub 2020 Jul 7. PMID: 32634339.

12: Chamuah JK, Dutta B, Borkotoky D. Pathological studies on helminth parasitic infection in mithun (<i>Bos frontalis</i>). J Parasit Dis. 2017 Dec;41(4):929-932. doi: 10.1007/s12639-017-0913-7. Epub 2017 Apr 20. PMID: 29114121; PMCID: PMC5660013.

13: Khan A, Ahmed H, Simsek S, Liu H, Yin J, Wang Y, Shen Y, Cao J. Molecular characterization of human Echinococcus isolates and the first report of E. canadensis (G6/G7) and E. multilocularis from the Punjab Province of Pakistan using sequence analysis. BMC Infect Dis. 2020 Apr 3;20(1):262. doi: 10.1186/s12879-020-04989-6. PMID: 32245373; PMCID: PMC7118937.

14: Goja S, Saha SK, Yadav SK, Tiwari A, Soin AS. Surgical approaches to hepatic hydatidosis ranging from partial cystectomy to liver transplantation. Ann Hepatobiliary Pancreat Surg. 2018 Aug;22(3):208-215. doi: 10.14701/ahbps.2018.22.3.208. Epub 2018 Aug 31. PMID: 30215042; PMCID: PMC6125266.

15: Korhonen PK, Kinkar L, Young ND, Cai H, Lightowlers MW, Gauci C, Jabbar A, Chang BCH, Wang T, Hofmann A, Koehler AV, Li J, Li J, Wang D, Yin J, Yang H, Jenkins DJ, Saarma U, Laurimäe T, Rostami-Nejad M, Irshadullah M, Mirhendi H, Sharbatkhori M, Ponce-Gordo F, Simsek S, Casulli A, Zait H, Atoyan H, de la Rue ML, Romig T, Wassermann M, Aghayan SA, Gevorgyan H, Yang B, Gasser RB. Chromosome-scale Echinococcus granulosus (genotype G1) genome reveals the Eg95 gene family and conservation of the EG95-vaccine molecule. Commun Biol. 2022 Mar 3;5(1):199. doi: 10.1038/s42003-022-03125-1. PMID: 35241789; PMCID: PMC8894454.

16: Khuroo MS, Datta DV, Khoshy A, Mitra SK, Chhuttani PN. Alveolar hydatid disease of the liver with Budd-Chiari syndrome. Postgrad Med J. 1980 Mar;56(653):197-201. Doi 10.1136/pgmj.56.653.197. PMID: 7393812; PMCID: PMC2425836.

17: Singh S, Khichy S, Singh M, Gill JS. Recurrent solitary hydatid cyst of the subcutaneous tissue. Indian J Surg. 2009 Jun;71(3):162-4. doi: 10.1007/s12262-009-0044-1. Epub 2009 Jun 10. PMID: 23133143; PMCID: PMC3452473.

18: Kowalczyk M, Kurpiewski W, Zieliński E, Zadrożny D, Klepacki Ł, Juśkiewicz W, Lasocki J, Dyśko Ł, Batia K, Pesta W. A rare case of the simultaneous location of Echinococcus multilocularis in the liver and the head of the pancreas: case report analysis and review of literature. BMC Infect Dis. 2019 Jul 24;19(1):661. doi: 10.1186/s12879-019-4274-y. PMID: 31340769; PMCID:PMC6657101.

19: Bajpai J, Jain A, Kar A, Kant S, Bajaj DK. "Necklace in the lung:" Multilocularis hydatid cyst mimicking left-sided massive pleural effusion. Lung India. 2019 Nov-Dec;36(6):550-552. doi: 10.4103/lungindia.lungindia_76_19. PMID: 31670306; PMCID: PMC6852218.

20: Bansal N, Vij V, Rastogi M, Wadhawan M, Kumar A. A report on three patients with Echinococcus multilocularis: Lessons learned. Indian J Gastroenterol. 2018 Jul;37(4):353-358. doi: 10.1007/s12664-018-0860-y. Epub 2018 Aug 18. PMID: 30121887.

21: Matossian RM, Rickard MD, Smyth JD. Hydatidosis: a global problem of increasing importance. Bull World Health Organ. 1977;55(4):499-507. PMID: 74294; PMCID: PMC2366677.

22: Talwar N, Agarwal N, Chugh K. A Unique Case of Cardiac Echinococcus multilocularis. Indian Pediatr. 2020 Dec 15;57(12):1181-1182. doi: 10.1007/s13312-020-2076-3. PMID: 33318327; PMCID: PMC7781830.

23: Tyagi DK, Balasubramaniam S, Sawant HV. Primary calcified hydatid cyst of the brain. J Neurosci Rural Pract. 2010 Jul;1(2):115-7. doi: 10.4103/0976-3147.71729. PMID: 21808518; PMCID: PMC3139339.

24: Laurimäe T, Kinkar L, Moks E, Romig T, Omer RA, Casulli A, Umhang G, Bagrade G, Irshadullah M, Sharbatkhori M, Mirhendi H, Ponce-Gordo F, Soriano SV, Varcasia A, Rostami-Nejad M, Andresiuk V, Saarma U. Molecular phylogeny based on six nuclear genes suggests that Echinococcus granulosus sensu lato genotypes G6/G7 and G8/G10 can be regarded as two distinct species. Parasitology. 2018 Dec;145(14):1929-1937. doi: 10.1017/S0031182018000719. Epub 2018 May 21. PMID: 29781421.

25: Huang J, Wu YM, Liang PC, Lee PH. Alveolar hydatid disease causing total occlusion of the inferior vena cava. J Formos Med Assoc. 2004 Aug;103(8):633-6. PMID: 15340664.

26: Gandhiraman K, Balakrishnan R, Ramamoorthy R, Rajeshwari R. Primary Peritoneal Hydatid Cyst Presenting as Ovarian Cyst Torsion: A Rare Case Report. J Clin Diagn Res. 2015 Aug;9(8):QD07-8. doi: 10.7860/JCDR/2015/14324.6397. Epub 2015 Aug 1. PMID: 26436004; PMCID: PMC4576597.

27: Kushwaha JK, Sonkar AA, Verma AK, Pandey SK. Primary disseminated extrahepatic abdominal hydatid cyst: a rare disease. BMJ Case Rep. 2012 May 30;2012:bcr0220125808. Doi 10.1136/bcr.02.2012.5808. PMID: 22669859; PMCID: PMC4543121.

28: Mohanty MC, Ravindran B. Deficiency of antibody responses to T-independent antigens in gerbils---Meriones unguiculatus. Dev Comp Immunol. 2002 May;26(4):385-91. doi: 10.1016/s0145-305x(01)00086-6. PMID: 11888652.

29: Prabhakar N, Kalra N, Behera A, Das A, Lal A, Dhiman RK, Khandelwal N. Large Heterogeneous Calcified Masses in Liver: A Diagnostic Dilemma. J Clin Exp Hepatol. 2017 Dec;7(4):385-386. doi: 10.1016/j.jceh.2017.09.001. Epub 2017 Oct 3. PMID: 29234206; PMCID: PMC5719463.

30: Aikat BK, Bhusnurmath SR, Cadersa M, Chhuttani PN, Mitra SK. Echinococcus multilocularis infection in India: The first case report was proved at an autopsy. Trans R Soc Trop Med Hyg. 1978;72(6):619-21. doi: 10.1016/0035-9203(78)90015-9. PMID: 734718.

31: Shaw AK, Gambhir RP, Chaudhry R, Jaiswal SS. Echinococcus multilocularis causing alveolar hydatid disease liver: a rare occurrence in the Indian subcontinent. Trop Gastroenterol. 2010 Ap Jun;31(2):119-20. PMID: 20862989.

32: Taneja K, Gothi R, Kumar K, Jain S, Mani RK. Peritoneal Echinococcus multilocularis infection: CT appearance. J Comput Assist Tomogr. 1990 May-Jun;14(3):493-4. PMID: 2335629.

Table 4A: Articles retrieved by expanded literature search.
1. Yattoo GN, Ahmad G, Rasool Z, Gulzar GM, Sodhi JS, Choh NA, et al. Abstract 017 Alveolar echinococcosis of liver: A series of ten cases. . 59th Annual Conference of Indian Society of Gastroenterology ISGCON 2018, KOCHI; November 29-December 1, 2018; Kochi, Kerala, India. 2018.<br><br>2. Mir SA, Mushtaq A, Intikhab M, Wani M. Clinical profile, diagnostic challenge, and management of alveolar hydatid disease: a prospective study. International Journal of Research in Medical Sciences. 2019;7(5):1500.<br><br>3. Ahmad I, Ilyas M, Ashraf A, Rather A, Gojwari T. Prevalence of hepatic alveolar hydatid in a nonendemic region of North India in hospital-based population: Emerging trend or improved diagnostic workup. Journal of Datta Meghe Institute of Medical Sciences University. 2021;16(1):86-9.<br><br>4. Parry AH, Wani AH, Feroz I. The spectrum of multimodality imaging findings in hepatic alveolar echinococcosis and the potential role of diffusion-weighted imaging in its characterisation. Pol J Radiol. 2020;85:e613-e23.<br><br>5. Vijay K, Vijayvergia V, Saha A, Naidu C, Rao P, Godara R. Hepatic alveolar hydatidosis—A malignant masquerade. Hellenic Journal of Surgery. 2013;85(2):135-8.

Table 5A. Duplicates of 79 articles were retrieved from PubMed on 10 May 2024. There were 35 duplicates, but the actual list was only 44 articles. Five articles retrieved by expanded additional search are included at the end, and none were duplicated.

Number	Articles from Table A1	Articles from Table 2A	Articles from Table 3A
	**Alveolar echinococcosis & India (n=19).**	**Echinococcus multilocularis & India (n=23)**	**Alveolar hydatid cyst & India (n=32)**
1		1. Bhalla 2023	2. Bhalla
2		2.Srinivas 2016	3.Srinavas 2016
3		3. Talwar 2020	10. Talwar 2020
4	11.Bansal 2018	4.Bansal 2018	19.Bansal 2018
5	1.Mitra 2024	5.Mitra 2024	4.Mitra 2024
6	9. Khan 2020	6. Khan 2020	25. Khan 2010
7	6.Dudha 2018	7.Dudha	8.Dudha 2018
8		8. Kowalczyk 2019	29.Kowalczk 2019
9	10.Korhonen 2022	9.Korhonen 2022	12.Korhonen 2022
10		10. Kumar 2023	6. Kumar 2023
11		11. Prabhakar 2017	22. Prabhakar 2017
12		12. Datta 2022	5.Datta 2022
13		13. Matossian 1977	20.Matossian 1977
14		14. Aikat 1978	31.Aikat 1978
15	12.Bajpai 2019	15.Bajpai 2019	11. Bajpai 2019
16		16. Mohanty 2002	26. Mohanty 2002
17		17. Kanoji 2020	13.Kanoji 2020
18		18. Gardhirman 2015	15.Gandhiraman 2015
19		19. Kushwaha 2012	24. Kushwaha 2012
20	15. Singh 2009	20. Singh 2009	15. Singh 2009
21		21. Taneja 1990	32.Taneja 1990
22		23. Shaw 2010	30. Shaw 2010
23	2.Dietrich 2020		7. Dietrich 2020
24	3. Jha 2015		
25	4.Madhsudhan 2016		
26	5. Parry 2020		17. Parry 2020
27	7. Wang 2009		
28	8. Goja 2018		23. Goja 2018
29	13.Kinkar 2018		
30	14. Kinkar 2018		

31	16. Kinkar 2018		
32	17. Tyagi 2010		21. Tyagi 2010
33	18.Laurimae2018		27. Laurinae 2018
34	19. Madhusudan 2015		
35			1. Bhatia 2016
36			9. Khuroo 1980
37			14. Chamuah 2017
38			18. Chaudhari 2017
39			28. Huang 2004
	**Five Articles retried from expanded additional search**		
40	Yattoo et al. 2018		
41	Ahmad et al. 2021		
42	Jahangir et al. 2020		
43	Mir et al. 2019		
44	Vijay et al. 2013		

Table 6A. Systematic Literature Review of published articles. The purpose was to determine the number of Alveolar echinococcosis cases reported in India. Search Engine: PubMed, articles. MeSH Terms: Alveolar echinococcosis and India (N=19 articles, Sheet 1 column), Echinococcus multilocularis and India (N+23 articles, Sheet 2 column), Alveolar hydatid and India (N=32, Sheet 3 column). The last 4 rows enlist articles found by the additional search.							
Serial number	**Articles for inclusion criteria. The list generated from Sheet 4**	Subject of study	Patients group [AE or others]	Author nationality	Patients' nationality	Region	# cases AE
1.	1 Bhalla et al. 2023	Review					
2.	2 Srinivas 2016	Review					
3.	3. Talwar 2020	Case study	AE	Indian	Indian	India	1
4.	4. Bansal 2018	Case study	AE	Indian	Kyrgyzstan	-	3
5.	5.Mitra 2024	Case study	AE	Indian	Indian	Kashmir	1
6.	6. Khan 2020	Genetic studies CE					
7.	7.Dudha	Case study	AE	Indian	Peru	-	1
8.	8. Kowalczyk 2019	Case study	AE	Poland	Poland		
9.	9.Korhonen 2022	Genetic studies CE					
10.	10. Kumar 2023	Case study	CE (Multicystic)				
11.	11. Prabhakar 2017	Case study	AE	Indian	Indian	Kashmir	1
12.	12. Datta 2022	Case study	CE (Multicystic)				
13.	13. Matossian 1977	Review					
14.	14. Aikat 1978	Case study	AE	Indian	Indian	Kashmir	1
15.	15 Bajpai 2019	Case study	CE (Multicystic)				
16.	16. Mohanty 2002	Immunity studies					
17.	17. Kanoji 2020	Case study	CE (Multicystic)				
18.	18. Gardhirman 2015	Case study	CE (Multicystic)				
19.	19. Kushwaha 2012	Case study	CE (Multicystic)				
20.	20. Singh 2009	Case study	CE				
21.	21. Taneja 1990	Case study	AE	Indian	Indian	Delhi	1
22.	23. Shaw 2010	Case study	AE	Indian	Indian	Soldier	1
23.	2.Dietrich 2020	Review					
24.	3. Jha 2015	Case study	AE	Indian	Kyrgyzstan		1
25.	4.Madhsudhan 2016	Review					
26.	5. Parry 2020	Case study	AE	Indian	Indian	Kashmir	23
27.	7. Wang 2009	Case study	AE	Chinese	Chinese		
28.	8. Goja 2018	Case study	AE	Indian	Kyrgyzstan	-	4
29.	13.Kinkar 2018	Review					

30.	14.Kinkar 2018	Genetic studies CE AE					
31.	16.Kinkar 2018	Genetic studies CE AE					
32.	17. Tyagi 2010	Case study	AE	Indian	Indian	Maharashtra	1
33.	18.Laurimae2018	Genetic studies CE AE					
34.	19. Madhusudan 2015	Review					
35.	1. Bhatia 2016	Case study	AE	Indian	Indian	Soldier	1
36.	9. Khuroo 1980	Case study	AE	Indian	Indian	Kashmir India	1
37.	14. Chamuah 2017	Pathological studies					
38.	18. Chaudhari 2017	Veterinary studies					
39.	28. Huang 2004	Case study	AE	Chinese	Chinese		
Articles based on additional search:							
40.	Yattoo et al. 2018	Case series	AE	India	India	Kashmir	10
41.	Ahmad et al. 2021	Case series	AE	India	India	Kashmir	25
42.	Jahangir et al. 2020	Case series	AE	India	India	Kashmir	6
43.	Mir et al. 2019	Case series	AE	India	India	Kashmir	23
44.	Vijay et al 2013	Case report	AE	India	India	-	1
Articles dealing with case reports, case series, and case studies of patients with AE from India were included.							
Shaded rows denote that the study did not fulfill the inclusion criteria for systematic review.							
Each study is shown as the first author with a year of publication. The number before each author shows the serial number in the publication in the search engine for that MeSH term.							

Table 7A. A systematic review of studies on patients with *Alveolar echinococcosis* published from India.

Author yr.	Dept. Institution	Study period	Study protocol	# cases	Residence	Age (yr.). Gender	Liver disease		Serology**	Stage (SI - SIV) # cases	Surgeries
							Lobe # cases	Size cm			
Khuroo et al. 1980	Hepatology PGIMER Chandigarh India	June 1977	Case report	1	Kashmir India	29 M	RL	14 x 18	P 1	SIV	Surgery, Died
Taneja et al. 1990	MRC&H, New Delhi, India.	1990	Case report	1	India (region not known)	60 M	Peritoneum & spleen	_	_	SIV	Splenectomy
Shaw et al. 2010	Gastroenterology AFMC Pune India	2010	Case report	1	Indian Soldier	31 M	RL	_	P1	_	Aspiration, Excision
Tyagi et al. 2010	Neurosurgery TNMC & BYL Nair Hospital Mumbai, India	2010	Case report	1	India (region not known)	25 M	Brain	_	_	_	Resection
Vijay et al 2013	GI Surgery Army Hospital New Delhi India	2013	Case report	1	Indian soldier	43 M	RL 6, 5, 7 & 8	7.4 x 6.6 x 7.9	-	SIV	-
Jha et al. 2015	Pathology, Medanta Medicity Gurgaon India	2015	Case report	1	Kyrgyzstan	31 F	RL, LL	_	_	SIV	Liver transplantation
Madhusudhan et al. 2015	Surgery AIIMS New Delhi, India	2016	Case report	1	India (region not known)	45 M	_	_	_	_	Resection
Bhatia et al. 2016	AFMC, Pune, India	2016	Case report	1	Indian Soldier	37 M	LL/RL	17	_	_	Left hepatectomy
Prabhakar et al. 2017	PGIMER, Chandigarh India	2017	Case report	1	India (region not known)	27 F	RL	14	_	_	_
Bansal et al. 2018	Fortis, Okhla New Delhi, India	July 2018	Case series	3	Kyrgyzstan	_	_	_	_	_	_
Goja et al. 2018	Liver Transplant, Medanta, Gurgaon, India.	Prospective study March	All cases of echinococcosis (n=25)	4	Central Asian Countries	33.7 ± 3.1 M1:F3	Liver	_	P 4	SIV 4	Liver transplantation 3, Tri-

		2010 to May 2016									segmentectomy 1.
Dudha et al. 2018	SMIMER Surat Gujrat India	2018	Case report	1	Peruvian islands, Peru	28 F	Lung	45 x 3.7	P	-	Resection
Yattoo et al. 2018	Gastroenterology SKIMS Srinagar Kashmir India	Three years (2016 to 2018)	Patients referred with SOL* liver	10	Kashmir India	39.0±11.0. M 5: F 5;	RL 8, LL 2	3.5 to 15	P 7	SI 2, SII 4, SIII 3, SIV 1	ERCP 3
Mir et al. 2019	Surgery, GMC Srinagar Kashmir India	Prospective study SMHS£ Hospital (June 2012 to Dec 2017)	Cases	13	Kashmir India	42.07±8.88 M5: F8	RL 2, LL 2, RL/LL 9	-	-	-	Resection 8, aspiration 1, unresectable 4
Talwar et al. 2020	Pediatric Pulmonology, Fortis, Gurgaon India.	2020	Case report	1	Iraq	7 F	RL/LL	_	P 1	SIV	Died two mon follow up
Jehangir et al. 2020	Radiodiagnosis GMC Srinagar Kashmir India	Jan 2017 to May 2019	Patients referred for imaging	6	Kashmir India	32.8±11.2. M 2: F 4	RL 4, LL 2	5.9 to 9.6	P 1, N 3	SII 5, SIV 1	Resections 2
Parry et al. 2020	Radiodiagnosis SKIMS & GMC Srinagar Kashmir India	Oct 2017 to Sept 2019	Patients referred for imaging	23	Kashmir India	_	Liver	_	_	SI 7, SI I5, SIII 7, SIV 4	_
Ahmad et al. 2021	Surgery SKIMS-MC Srinagar Kashmir India	April 2017 to March 2019	Patients referred for ultrasound [n=120, 620]	25	Kashmir India	53.4 (30 to 70). M 12: F 13	Liver	_	_	_	_
Mitra et al. 2024	Surgery PGIMER Chandigarh, India	2024	Case report	1	Kashmir India	36 M	RL	12.5	_	_	Resection
Khuroo et al. (present study)	Dr. Khuroo's Medical Clinic	Prospective Study 2005 to 2024	All cases of echinococcosis [n=411]	12	Kashmir India	46.6±11.9. M 4:F 8	RL 11, LL 3, RL/LL 1	3.5 to 16.5	P 12	SI 3, SIIIb 3, SIV 6	Surgery 4, Aspiration 1

	Srinagar Kashmir India										
Systematic literature review	Several Clinical, Imaging & Pathology Units in Kashmir, India	Sept 2019 to April 2024	Personal inquiries	38	Kashmir India 27, West Bengal India 1, Central Asia 10	_	Liver	_	_	_	LT6, Resections11_

PGIMER =Post-graduate Institute of Medical Education and Research, MRC&H =Medical Research Centre and Hospital, GGSMC= Guru Gobind Singh Medical College, AFMC = Armed Forces Medical College, TNMC & BYL Nair Hospital= Topiwala National Medical College And Bai Yamunabai Laxman Nair Charitable Hospital, Mumbai, SMIMER= Surat Municipal Institute of Medical Education and Research, GMC =Govt. Medical College, SKIMS =Sher-I-Kashmir Institute of Medical Sciences, SKIMS-MC =SKIMS Medical College, £SMHS=Shri Maharaja Hari Singh Hospital, KGMC=King George Medical College, *SOL=space occupying lesion, **P=P stands for number of patients with positive echinococcal serology, N stands for those with negative serology,

REFERENCES FOR SYSTEMATIC REVIEW

1. Page MJ, McKenzie JE, Bossuyt PM, Boutron I, Hoffmann TC, Mulrow CD, et al. The PRISMA 2020 statement: an updated guideline for reporting systematic reviews. BMJ. 2021;372:n71.
2. PRISMA Extension for Scoping Reviews (PRISMA-ScR): Checklist and Explanation. Annals of Internal Medicine. 2018;169(7):467-73.
3. Khuroo MS, Khuroo NS, Khuroo MS. Accuracy of Rapid Point-of-Care Diagnostic Tests for Hepatitis B Surface Antigen-A Systematic Review and Meta-analysis. J Clin Exp Hepatol. 2014;4(3):226-40.
4. Khuroo MS, Khuroo NS, Khuroo MS. Diagnostic Accuracy of Point-of-Care Tests for Hepatitis C Virus Infection: A Systematic Review and Meta-Analysis. PLOS ONE. 2015;10(3):e0121450.
